# Characterization of Multi-Functional Properties and Conformational Analysis of MutS2 from *Thermotoga maritima* MSB8

**DOI:** 10.1371/journal.pone.0034529

**Published:** 2012-04-24

**Authors:** Euiyoung Jeong, Hunho Jo, Tae Gyun Kim, Changill Ban

**Affiliations:** Department of Chemistry, Pohang University of Science and Technology, Pohang, Gyungbuk, South Korea; University of Massachusetts Medical School, United States of America

## Abstract

The MutS2 homologues have received attention because of their unusual activities that differ from those of MutS. In this work, we report on the functional characteristics and conformational diversities of *Thermotoga maritima* MutS2 (TmMutS2). Various biochemical features of the protein were demonstrated via diverse techniques such as scanning probe microscopy (SPM), ATPase assays, analytical ultracentrifugation, DNA binding assays, size chromatography, and limited proteolytic analysis. Dimeric TmMutS2 showed the temperature-dependent ATPase activity. The non-specific nicking endonuclease activities of TmMutS2 were inactivated in the presence of nonhydrolytic ATP (ADPnP) and enhanced by the addition of TmMutL. In addition, TmMutS2 suppressed the TmRecA-mediated DNA strand exchange reaction in a TmMutL-dependent manner. We also demonstrated that small-angle X-ray scattering (SAXS) analysis of dimeric TmMutS2 exhibited nucleotide- and DNA-dependent conformational transitions. Particularly, TmMutS2-ADPnP showed the most compressed form rather than apo-TmMutS2 and the TmMutS2-ADP complex, in accordance with the results of biochemical assays. In the case of the DNA-binding complexes, the stretched conformation appeared in the TmMutS2-four-way junction (FWJ)-DNA complex. Convergences of biochemical- and SAXS analysis provided abundant information for TmMutS2 and clarified ambiguous experimental results.

## Introduction

Prokaryotic and eukaryotic MutS homologues have been widely known as central enzymes in the DNA methyl-directed mismatch repair (MMR) mechanism [1]–[4]. Various studies indicate that eukaryotic MutS homologues (MSHs) are associated with not only post-replication repair of mispaired lesions but also meiosis-specific DNA recombination and DNA repair mechanisms [1], [5], [6]. In addition, bacterial MutS homologues have been continuously investigated because of their diverse functions [7]. Particularly, bacterial MutS2 has attracted much interest because of its high sequence homology with a eukaryotic MSH4-MSH5 heterodimer [8], [9] that plays an important role in meiotic crossing-over and recombination [6], [10], [11]. Furthermore, sequence analysis between the MutS and MutS2 homologues implies that the MutS2 protein may have potential functions that are not shared with MutS [9], [12]. For example, bacterial MutS2 homologues have shown that the deletion of the *Helicobacter pylori* MutS2 gene *in vivo* results in increases of DNA recombination, indicating that the protein suppresses homologous and homeologous DNA recombination [13]–[15].

It has been known that bacterial MutS2 contains a hydrolytic ATPase and possesses a selective binding affinity for DNA substrates but no specificity for homoduplex or heteroduplex DNA [16]–[20]. In addition, it includes a small-mutS related (Smr) domain with a non-specific nicking endonuclease function [12], [17]–[22]. In particular, this Smr domain is spread widely throughout many species in such proteins as the BCL-3-binding protein (B3bp) in humans [23], [24], the GUN1 protein in plants [25], and the YdaL and YfcN proteins in *Escherichia coli* [21], [26]. Although the ATPase and Smr domains are present in a large variety of prokaryotes and eukaryotes, in the case of MutS2, the biological functions as well as the functional correlations between the MutS2 domains and other homologues have not been clearly elucidated. Additionally, the conformational changes of the ATPase and Smr domains caused by binding nucleotides and DNA must be clarified. Therefore, it is necessary to study the functional characteristics and structural features of MutS2 in detail to understand the valuable functions of the protein.

Small-angle X-ray scattering (SAXS) is one of the effective tools for analyzing the solution structures of bio-macromolecules, and offers various pieces of information such as domain organization, intermolecular interaction, and large-scale structure. Through the analysis of the scattering pattern at very small angles using a direct X-ray beam, the conformations of the samples can be obtained on a scale between 1 nm and several hundred nanometers [27]. This technique is readily applicable to the observation of the conformations of many biomolecules, including DNA, RNA, and proteins [28]. Hence, the structural features of partial domains in a protein can be easily and effectively confirmed using the SAXS method.

In this study, we investigated the biochemical properties and solution structures of the MutS2 protein from a hyper-thermophilic bacterium, *Thermotoga maritima* MSB8, which was extracted from geothermal-heated marine sediment [29]. The nicking endonuclease activity of *T. maritima* MutS2 (TmMutS2) was verified by various biochemical assays, and the effects of temperature, cations, DNA, and nucleotides on endonuclease activity were examined. Additionally, the relationships between TmMutS2 and *T. maritima* MutL (TmMutL) or *T. maritima* RecA (TmRecA) were demonstrated using both *in vitro* and *in vivo* assays. In addition, the conformational transitions of TmMutS2 in the presence of nucleotides and DNA were confirmed using biochemical and SAXS modeling methods. Superimpositions of the TmMutS2 SAXS models onto the crystal structures of B3bp-Smr and the ATPase domain of *Thermus aquaticus* MutS (TaqMutS) indicated a resemblance between the SAXS models and the crystal structures.

## Results

### Characterization of Biochemical Properties for TmMutS2

Prior to the verification of characteristics for TmMutS2, its sequence was analyzed via comparisons with homologues through BLAST searches (Figure S1). Sequence alignment analysis indicated common features between TmMutS2 and other MutS2 homologues via the conserved DNA-binding and ATP-hydrolytic domains (Figure S1). TmMutS2, as well as other MutS2 homologues such as *H. pylori* MutS2, *E. coli* YdaL, and *Thermus thermophilus* MutS2 [13], [17]–[19], contain a conserved C-terminal domain of approximately 100 to 250 amino acid residues, which is identified as the Smr domain. They also lack a conserved N-terminal region as a recognition site for mismatched DNA, whereas *E. coli* MutS does not possess the Smr domain but does have the N-terminal recognition site. Therefore, it is expected that TmMutS2 has the Smr-related functions but lacks the identification activity for mismatched DNA.

A highly conserved Walker’s A-type nucleotide-binding motif in TmMutS2 (Figure S1) represents the ATPase domain as previously reported for *T. thermophilus* MutS2 [17]. To examine the ATPase activity of TmMutS2, an ATPase assay was carried out in two ways using highly purified TmMutS2 (89 kDa, Figure S2). Polyethyleneimine-cellulose thin-layer chromatography (PEI-TLC) analysis revealed that TmMutS2 had a higher ATPase activity at 60°C compared to 37°C, and a fluorometric analysis also indicated a two-fold increase of activity at 60°C compared with 37°C (Figure S2). To clarify the effect of instability of TmMutS2, dynamic light scattering (DLS) measurements at both 37°C and 60°C with time dependence (0, 30, 60, and 90 min) were conducted. As shown in Figure S2, the average size of TmMutS2 was invariant despite variations of temperature and time. This indicates that the stability of the protein was maintained during the measurement of ATPase activity. Therefore, the ATPase activity discrepancy is only influenced by temperature. In addition, double-stranded DNA (dsDNA) slightly affects the ATP-hydrolytic activity of TmMutS2 as represented in Figure S2. These results indicate that TmMutS2 possesses thermo-active ATPase activity that functions in a DNA-dependent manner [17].

**Figure 1:**
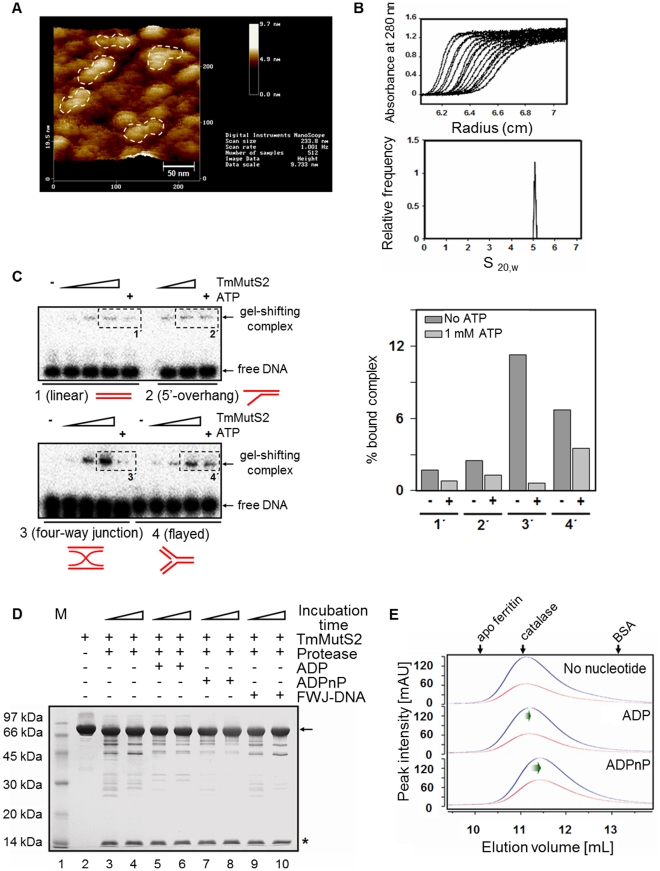
Biochemical properties of TmMutS2. (**A**) The SPM image of the TmMutS2 protein. Height is indicated by the colors dark (0 nm) and light (9.7 nm) brown. The shape of TmMutS2 molecules on the mica looks appears to be a two-fold symmetric form (the white dashed line). (**B**) Sedimentation velocity analysis of TmMutS2. The absorbance at 280 nm was recorded every 4 min (upper plot). The lower plot is the C(s) analysis of TmMutS2. (**C**) DNA binding activities of TmMutS2 in the absence or presence of ATP (1 mM). TmMutS2 concentration is varied from 0 to 2 µM. DNA substrates 1, 2, 3, and 4 are the linear, overhanging, four-way junction, and flattened linear flayed forms, respectively. The right plot indicates the % bound complex, represented by the dashed line boxes (1′–4′), in the gel. (**D**) Limited digestion by α-chymotrypsin. Lane 1 represents the protein molecular weight marker. Lane 2 is the protease-untreated TmMutS2 (5 µg). TmMutS2 was digested in the presence of 0.3 mM ADP (lanes 5–6), 1 mM ADPnP (lanes 7–8), and 1 µM FWJ-DNA (lanes 9–10). The arrow indicates the N-terminal truncated form (residue 117 to 757) and the asterisk marks the N-terminal fragment. (**E**) Size exclusion chromatographic analysis. Blue and red peaks are the detection profiles of absorbance at 280 and 254 nm, respectively. The molecular sizes of TmMutS2 and its complexes with nucleotides were estimated from their elution volumes (V_e_). The protein standards were used for the standardization of molecular weights using K_av_ = (V_e_–V_o_)/(V_c_–V_o_), where V_c_ is the geometric column volume and V_o_ is the void volume that is calculated by Blue dextran (2000 kDa).

The multimeric status of TmMutS2 was confirmed by two ways. In the scanning probe microscopy (SPM) observation, the shape of the TmMutS2 molecules is a two-fold symmetric form, implying that TmMutS2 may form a dimer (white dash line) as shown in Figure 1A. This dimeric status was further determined by analytical ultracentrifugation. The distribution of the sedimentation coefficient was obtained using the C(s) method [30], [31]. A single narrow peak with a mean average sedimentation coefficient of 5.2 (±0.2)×10^–13^ s indicated that TmMutS2 exists as a homogeneous dimer (Figure 1B).

It is well known that MutS2 homologues interact with various forms of DNA [15]–[17]. In order to ascertain the DNA binding activity of TmMutS2, gel electrophoresis was carried out. TmMutS2 more strongly bound to four-way junction (FWJ) and flayed DNA (a fork structure) compared to linear and overhang DNA (Figure 1C). The addition of 1 mM ATP decreased the gel-shifting yield of TmMutS2-DNA complexes (particularly FWJ-DNA), thus suggesting that ATP acted as an obstacle with DNA for binding with TmMutS2. In addition, super-shifted bands on the gel were not detected after adding ATP. Hence, it is obvious that nucleotides play a crucial role in the binding of DNA to TmMutS2 even absent any alteration of multimeric status of the complexes.

The binding of nucleotides may affect the conformation of the protein. Proteolytic digestion analysis by α-chymotrypsin indicated conformational transitions of the TmMutS2-nucleotide and -DNA complexes (Figure 1D). Only FWJ-DNA was selected for proteolytic digestion due to it’s the strongest binding affinity with TmMutS2 among several types of DNA. TmMutS2 with nucleotides (particularly nonhydrolytic ATP; ADPnP) revealed faint digested bands rather than apo or complex forms with FWJ-DNA, providing clear evidence that TmMutS2 underwent conformational changes in a nucleotides-activated manner. Size exclusion chromatographic (SEC) analysis was also employed to verify the effects of nucleotides on conformation. Because of insignificant differences of digested bands between apo and FWJ-DNA complex forms, TmMutS2-nucleotide complexes were only applied to SEC analysis. As represented in Figure 1E, the elution profile of the native TmMutS2 exhibited only one broad peak ranging from 160 to 190 kDa, implying a dimeric TmMutS2 without any aggregate form. The results support the SPM and sedimentation velocity analysis. However, the increased elution volumes, 11.13 mL for TmMutS2-ADP and 11.42 mL for TmMutS2-ADPnP complexes, were noted (compare with 11.05 mL for apo-TmMutS2). These results suggest that there is shrinkage of TmMutS2 after nucleotide binding. Therefore, we suggest that the structure of TmMutS2 assumed a compacted form in the presence of nucleotides, particularly non-hydrolyzable ADPnP.

### Identification of Nicking Endonuclease Activity for TmMutS2

**Figure 2:**
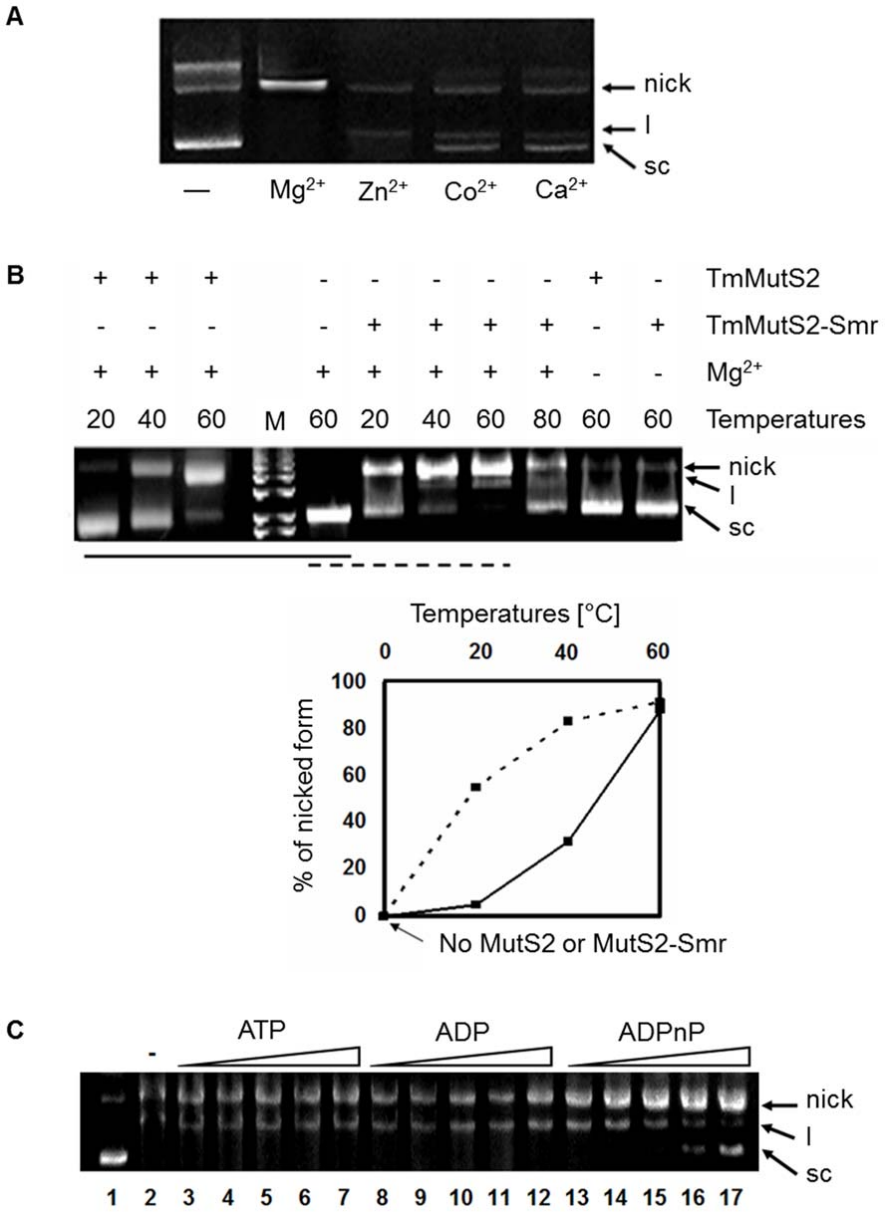
Nicking endonuclease activity of TmMutS2. (**A**) The effects of various cations on the nicking endonuclease activity of TmMutS2. The cations were used at a concentration of 10 mM. The nick, l, and sc indicate the nicked, linearized, and supercoiled open circular DNA forms, respectively. (**B**) A mixture containing 2 µM TmMutS2/TmMutS2-Smr and 1.5 µM plasmid DNA (linear size, 3 kbp) was incubated at temperatures ranging from 20°C to 80°C in the presence or absence of Mg^2+^ (upper plot). In the middle of the gel, M indicates the DNA size marker. The lower plot presents the percentage yields of the nicked form. (**C**) Different activities of DNA digestion in the presence of ATP (lanes 3 to 7), ADP (lanes 8 to 12), and ADPnP (lanes 13 to 17). Lane 1 and 2 are TmMutS2 untreated- and treated-plasmid DNA bands in the absence of theh nucleotide at 60°C. Each nucleotide was utilized at a final concentration of 3 mM.

It has been widely known that the nicking endonuclease activity of MutS2 homologues alters closed circular dsDNA leading to an open circular form under variable physical conditions [17], [18], [32]. Thus, it was expected that this TmMutS2 would also have inherent endonuclease activity. To demonstrate the DNA nicking endonuclease activity of TmMutS2, two types of protein, apo-TmMutS2 and TmMutS2-Smr, were incubated with freshly prepared plasmid DNA. These reactants were then analyzed by 0.8% agarose gel electrophoresis. First, various cations were applied to TmMutS2 for optimizing its nicking endonuclease activity (Figure 2A). The Mg^2+^ ion was determined to be the most effective cation to promote incision of plasmid DNA by TmMutS2, so it was applied to all reactions. In the absence of Mg^2+^, TmMutS2 presented lower nicking endonuclease activity (Figure 2B, right two lanes). This reaction was carried out in the buffer with 0.1 mM EDTA to eliminate trace of Mg^2+^. The nicking endonuclease activity of TmMutS2 increased in proportion to the tested temperature, ranging from 20°C to 60°C (left three lanes). In addition, TmMutS2-Smr also exhibited temperature-dependent endonuclease activity, but activity decreased in harsh conditions such as temperatures of 80°C (Figure 2B, from 6 th lane to 9 th lane). TmMutS2-Smr has higher activity compared to native TmMutS2 (Figure 2B, lower plot), indicating that endonuclease activity was confined to TmMutS2-Smr, which corresponded to the characteristics of other MutS2 homologues. A further experiment substantiated this fact as described in Figure S3. TmMutS2 and TmMutS2ΔSmr were incubated with supercoiled circular dsDNA (scDNA). TmMutS2ΔSmr presented no nicking endonuclease activity, suggesting that the Smr domain of TmMutS2 played a major role in the incision of the scDNA. TmMutS2-Smr, as well as human B3bp-Smr possessed a high level of nicking endonuclease activity, as expected (Figure S3). Therefore, it was anticipated that several species without endonucleases, such as MutH, possess alternative proteins that have the active Smr domain to provide the endonuclease function.

Nicking endonuclease activity was influenced by nucleotides (ATP, ADP, and ADPnP). As shown in Figure 2C, ATP and ADP did not affect the nicking endonuclease activity of TmMutS2, but quite high concentrations (above 2.5 mM) of ADPnP negatively impacted the plasmid DNA digestion, indicating the binding of ADPnP caused a decrease in the TmMutS2 activity. It was predicted that the nicking endonuclease activity of MutS2 proteins may be controlled by a particular nucleotide.

### Analysis of the MutL Effect on Endonuclease Activity of TmMutS2

**Figure 3:**
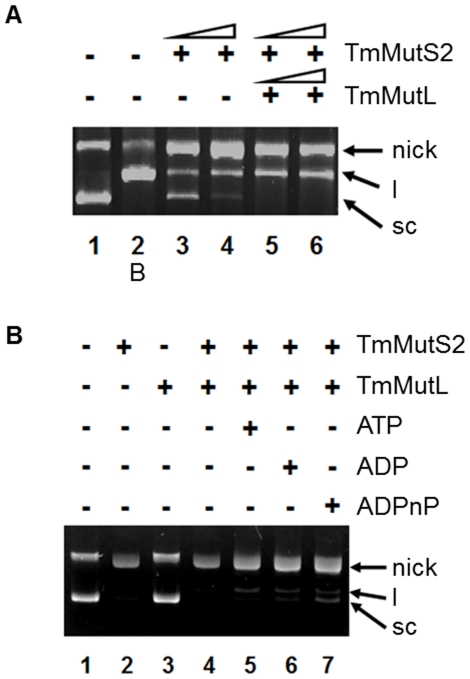
The TmMutL effects on the endonuclease activity of TmMutS2. (**A**) The 0.8% agarose gel indicates that TmMutL enhances nicking endonuclease activity and the DNA binding affinity of TmMutS2. Lane 1 represents TmMutS2 untreated DNA, and the B at lane 2 indicates EcoRI-treated linearized dsDNA. (**B**) The addition of 1 mM of nucleotide and 2 µM TmMutL to TmMutS2.

*E. coli* MutL is already known for participating in the MMR system [1], and MutL homologues from other bacterial species have been recognized as the activators for endonuclease activity of their respective MutS2 proteins [17]. The addition of TmMutL to TmMutS2 also resulted in an increase in nicking endonuclease activity (Figure 3A). This was in accordance with other evidence indicating that the TmMutS2-TmMutL complex has higher dsDNA binding affinity rather than TmMutS2 alone (Figure S4). Additionally, we examined the impact of nucleotides on the endonuclease activity of the TmMutS2-TmMutL mixture. The addition of nucleotides, particularly ADPnP, caused a decrease in the nicking endonuclease activity of TmMutS2 via the binding of the nucleotides to TmMutL (Figure 3B). As shown in Figure S5, only TmMutL did not show endonuclease activity as well as dependence on the nucleotide. These results suggest that the alterable nicking endonuclease activity of TmMutS2 is negatively controlled by the interaction of TmMutL with nucleotides.

### Confirmation of the Influences of TmMutS2 on DNA Homologous Recombination

**Figure 4:**
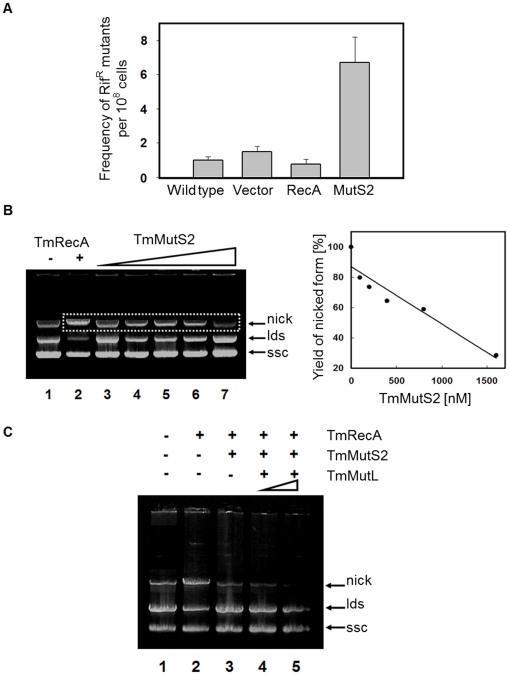
Suppression of the TmRecA-mediated DNA recombination reaction by TmMutS2. (**A**) A spontaneous mutation frequency assay using the incorporation of TmmutS2 gene into the *E. coli* BL21(DE3) strain. Rif^R^ indicates rifampicin-resistance. The frequency of Rif^R^ (y-axis)/10^8^ cells is quantitatively analyzed by counting the number of spontaneously mutated colonies. (**B**) The effect of the concentration of TmMutS2 on the TmRecA-mediated DNA recombination reactions. Lanes 2, 3, 4, 5, 6, and 7 correspond to 0, 100, 200, 400, 800, and 1600 nM of TmMutS2, respectively. Nick, lds, and ssc indicate the nicked, linearized, and supercoiled forms of open circular DNA, respectively. The right plot is the quantification of the nicked forms. This plot represents the changes of TmMutS2 concentrations versus the percentage yield of nicked-form DNA. (**C**) To observe the influence of TmMutL in the DNA strand exchange reaction, TmRecA, TmMutS2, and TmMutL were incubated at 37°C.

To understand the role of TmMutS2 on DNA homologous recombination, a spontaneous mutation frequency assay was carried out using TmRecA, a core protein involved in DNA strand exchange. As shown in Figure 4A, the mutation frequency of the BL21(DE3) strain with only pET-28a Tobacco Etch virus (TEV) and pET-28aTEV-TmrecA did not yield any significant difference in mutational frequency compared with the wild-type strain (1.39 (±0.03)×10^–8^ and 0.91 (±0.07)×10^–8^ rifampicin-resistance colonies, respectively), while incorporation of the *TmmutS2* gene into pET-28aTEV resulted in an approximately 6-fold increase of the spontaneous mutation frequency (6.63 (±0.13)×10^–8^). Additionally, the mutation on the Smr domain caused conspicuous decrease of the mutation frequency in comparison with wild type TmMutS2 (2.67 (±0.20)×10^–8^ and 7.33 (±0.47)×10^–8^ rifampicin-resistance colonies, respectively), indicating that the Smr domain plays an important role in the RecA-related recombination (Figure S6). This recombination experiment reveals that *TmmutS2* may affect the RecA-related recombination and act as a dominant mutation phenotype similar to other MutS2 homologues [15].

In order to inspect the effect of TmMutS2 on DNA homologous recombination in detail, TmMutS2 was incubated with TmRecA. RecA catalyzes extensive homoduplex formation between linearized DNA and single-stranded circular DNA (sscDNA) in the presence of single-stranded DNA-binding protein (SSB) and an ATP-regenerating system [33]. To further clarify the correlation between TmRecA and TmMutS2, TmMutS2 was applied to this system. Purified, closed dsDNA was completely linearized with EcoRI restriction enzyme for the apparent reaction between homologous M13 DNAs (linearized form and sscDNA). Then, linearized dsDNA (ldsDNA) and sscDNA were mixed into the reaction as described above. Faint band for the nicked form appeared in the control case due to the strand exchange reaction (Figure 4B, lane 1). The addition of TmRecA caused successful recombination between homoduplex DNA, indicating an increase in the nicked forms; new recombinant DNA consisted of sscDNA and one strand of ldsDNA (Figure 4B, lane 2). In the case of the TmMutS2 supplementation, the higher the concentration of TmMutS2, the vaguer the band of nicked dsDNA became (Figure 4B, lanes 3, 4, 5, 6, 7), indicating that TmMutS2 suppressed the TmRecA-mediated DNA strand exchange reaction. As shown in Figure 4B (right plot), the yields of nicked DNA linearly decreased with increases in TmMutS2 concentration. The addition of TmMutL resulted in the loss of nicked DNA compared to TmMutS2 alone (Figure 4C). Therefore, TmMutS2 effectively inhibited RecA-mediated DNA recombination, and TmMutL also interrupted the DNA strand exchange reaction. These results strongly suggest that TmMutL may play an important role in the regulation of TmMutS2-mediated recombination activity.

### Predictions of Structures using SAXS for TmMutS2 Complexes with Various Types of Nucleotides or DNA

**Figure 5:**
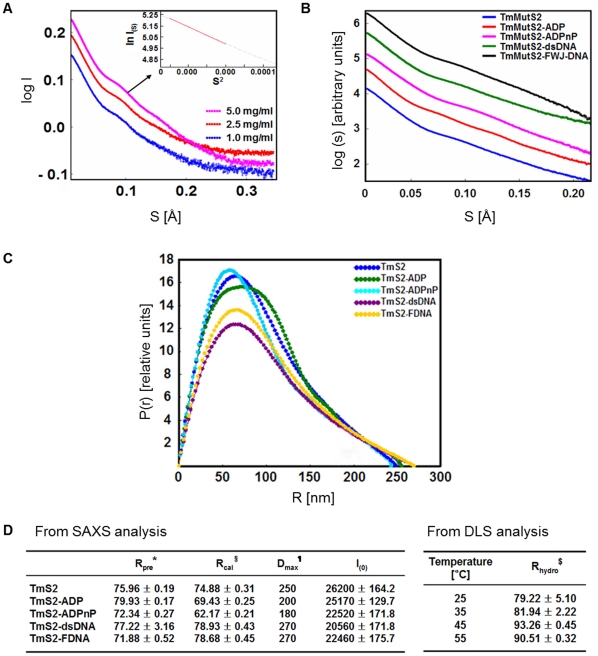
SAXS analysis for the structures of TmMutS2 and its complexes with nucleotides and DNAs. (**A**) SAXS scattering curves of apo-TmMutS2 at a concentration range of 1 to 5 mg/mL. The insert exhibits the Guinier approximation for the scattering data of TmMutS2 (5 mg/mL). (**B**) SAXS scattering curves of apo-TmMutS2 and its complexes with nucleotides (ADP and ADPnP) and DNAs (dsDNA and FWJ-DNA) as calculated by the PRIMUS program. (**C**) P(R) function plots estimated by GNOM program. (**D**) SAXS data parameters from a GNOM analysis and DAMMIN modeling. TmS2 is *T. maritima* TmMutS2. R_pre_^*^ was calculated from the Guinier approximation using AutoR_g_ program. R_cal_^§^ and D_max_^¶^ (the maximum diameter) were calculated from the P(r) function by GNOM program. FDNA represents four-way junction DNA. D_max_ is the longest distance of the SAXS models as determined by the DAMMIN program. Using DLS analysis, the hydrodynamic radii (R_hydro_^$^) were calculated at various temperatures.

For conformational analysis of proteins, SAXS can be directly applied to monitor the solution conformations of proteins [34], [35]. In this study, we collected SAXS data on the monodispersed solutions of TmMuS2 and its complexes with nucleotides (ADP and ADPnP) or DNA (linear dsDNA and FWJ-DNA). As shown in Figure 5A, the overlying scattering curves for the various concentrations of TmMutS2 indicate an increase in signal according to the concentration of TmMutS2, including some fluctuations at high S (Å). These signal fluctuations at the high-angle region result from structural features such as the total structure and the organization of domains [36]. Additionally, confirmation of the aggregated state of the protein can be provided by the scattering data, inspecting the linearity at the low S range using a Guinier approximation (lnI(S) vs. S^2^). The upper inset in Figure 5A presents the apo-TmMutS2, indicating that TmMutS exhibits monodispersity and no aggregation. For complexes of TmMutS2 with nucleotides and DNA, different signals were noted according to the partner molecules in the complex (Figure 5B). The Guinier approximation demonstrated that TmMutS2 complexes also displayed monodispersity and did not aggregate (Figure S7).

To obtain information about the structures of proteins from SAXS data, the P(R) spectra, histograms for the existence probabilities of atoms at all distances within scattering particles, were constructed (Figure 5C). From these spectra, the radius of gyration (R_g_) and the maximum particle dimension (D_max_) were calculated using the GNOM program and summarized in Figure 5D (left table). R_pre_ and R_cal_ represent the values estimated from the Guinier approximation and the P(R) spectrum, respectively, and R_cal_ was selected as the R_g_ value for further discussion. In the case of TmMutS2-dsDNA and TmMutS2-FWJ-DNA, the R_g_ and D_max_ were similar to those of apo-TmMutS2. However, the distance distributions of TmMutS2-ADP and TmMutS2-ADPnP were narrower than that of apo-TmMutS2 and had a smaller D_max_ than apo-TmMutS2. These results indicate that the addition of nucleotides to TmMutS2 induces a smaller protein compared to the apo-TmMutS2 form. Meanwhile, the calculated R_g_ of apo TmMutS2 was 74.88±0.31 Å, and D_max_ was 250 Å. The hydrodynamic radius (R_h_) was also calculated using DLS analysis in a temperature range of 25°C to 55°C. The calculation produced an averaged R_h_ value of 82.98 (±2.05) Å, indicating that TmMutS2 retains thermostability (Figure 5D, right table). In particular, the R_h_ at 45°C was 93.26 (±0.45) Å, exhibiting that the ratio of R_h_ to R_g_ is approximately 1.21, which is a similar result to the generally observed ratio (∼1.29) for known spherical molecules [37]. Thus, TmMutS2 may have a spherical configuration.

**Figure 6:**
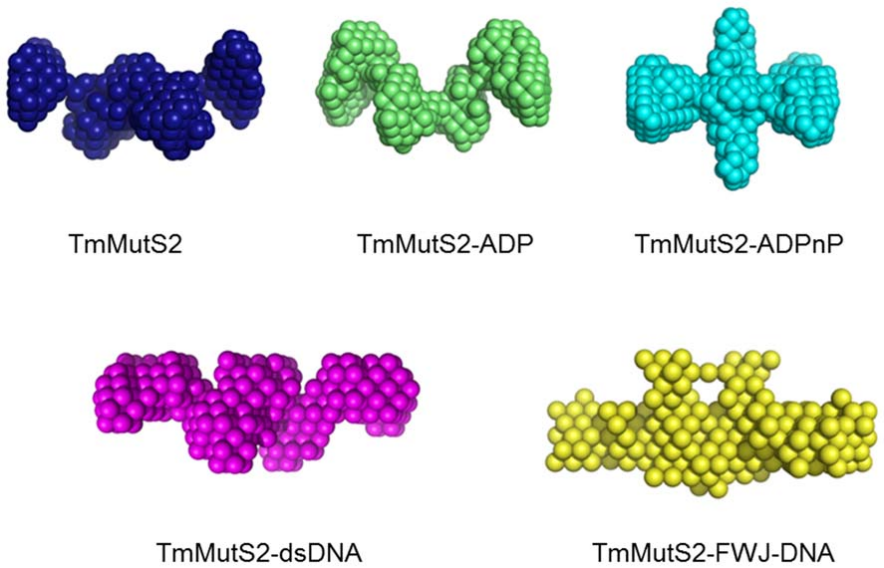
The best fitted *ab initio* SAXS models. They are depicted with space-filled dummy atoms.

Based on the scattering data, the structures for the TmMutS2 complexes were re-constructed using the *ab initio* modeling program DAMMIN (Figure 6). The SAXS models of apo-TmMutS2 and its complexes presented the two-fold symmetry, consistent with the results of SPM, SEC analysis, and the sedimentation velocity measurement that verified the homogeneous dimer conformation of TmMutS2 (Figure 1A, 1B, and 1E). In addition, apo-TmMutS2 and its complexed forms seem to have two or four linking domains based on the results of the SPM analysis of the surface-distributed TmMutS2 (size range 30 to 50 nm, Figure 1A). Additionally, the TmMutS2-ADP complex retained the slightly reduced size and small valley in the central region. Unlike apo-TmMutS2 and the TmMutS2-ADP complex, the large conformational changes in TmMutS2-ADPnP were observed in the central region. The compacted TmMutS2-ADPnP complex had the smallest R_g_ at 62.17 (±0.21 Å) and a D_max_ of 180 Å, as described in above. The compactness of TmMutS2-ADPnP was already proven by the SEC analysis (Figure 1E). The TmMutS2-dsDNA and TmMutS2-FWJ-DNA complexes exhibited lengthened structures compared to the apo-TmMutS2 form. In particular, TmMutS2-FWJ-DNA formed an elongated structure resulting from large conformational changes, indicating that intermolecular interactions between the protein and FWJ-DNA may create a nearly globular structure. These results suggest that TmMutS2 assumes a flexible conformation in the presence of nucleotides and DNA.

### Superimposition of the ATPase and Smr Models of TmMutS2 to the SAXS Models

**Figure 7:**
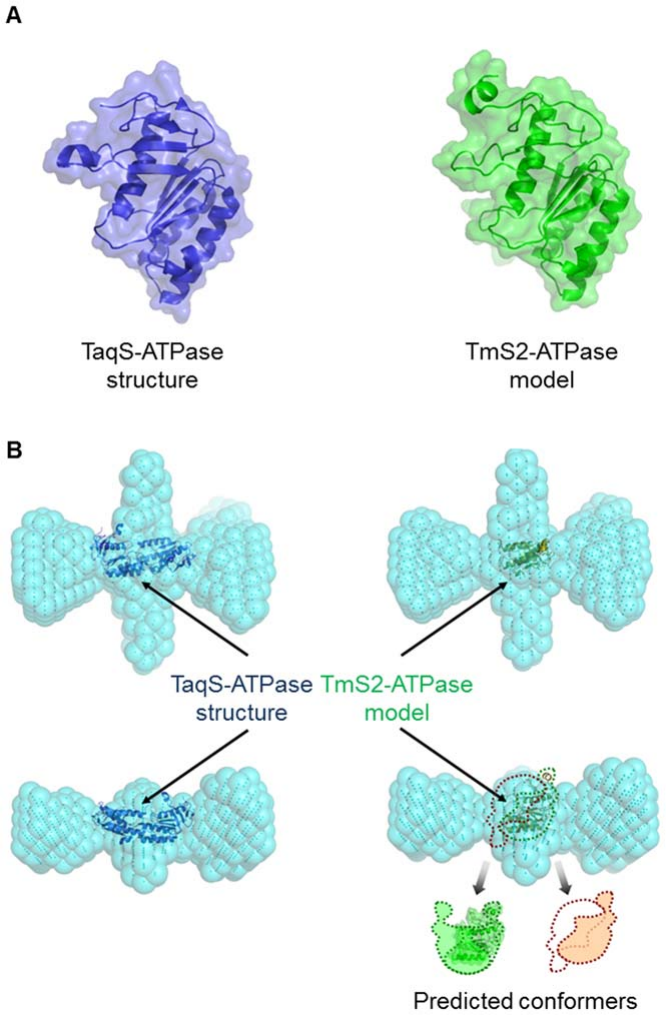
Superimpositions between the TmMutS2-ADPnP SAXS model and the ATPase structure/model. (**A**) The left structure is the ATPase domain of *T. aquaticus* MutS (TaqS-ATPase), and the right model is the predicted monomeric ATPase domain of TmMutS2 (TmS2-ATPase). (**B**) Superimpositions of the TmMutS2-ADPnP model to the dimeric TaqS-ATPase structure and the TmS2-ATPase model. The predicted conformers indicate the feasible structures of the dimeric TmS2-ATPase model.

Although much effort has been expended to define the crystal structure of TmMutS2, no one has done so. Therefore, to confirm the validity of our SAXS models, other structural information about TmMutS2 was required. As shown in Figure S8, the ATPase domain of TmMutS2 was aligned with TaqMutS. The alignment indicates that the secondary structures of the two proteins have similar features. Hence, an ATPase domain model of TmMutS2 computationally predicted from a crystal structure of TaqMutS (Protein Data Bank, PDB No. 1EWQ) was utilized as a template for structural overlapping instead of a whole crystal structure of TmMutS2 (Figure 7A). The solution SAXS models of the apo- and ADP-complexed forms could not be superimposed onto the dimeric ATPase domain of TaqMutS and the monomeric TmMutS2 because they may not form compacted structures. However, the compacted TmMutS2-ADPnP complex model was suitable for superimposition of the ATPase domains to the SAXS model (Figure 7B). In addition, whereas there is variability in the superimposition method using symmetrical differences [38], the normalized spatial discrepancy (NSD) algorithm enables fast superimposition of structural models and provides for quantitative analysis of the similarity between the models. Like error-weighted averaging discrepancy [39], NSD is characterized by fineness parameters that play the role of standard deviations for one-dimensional data sets. As shown in Table S1, the final fineness values for the superimposed SAXS structures were lower than their initial values. These tendencies make it more reliable the SAXS models.

**Figure 8:**
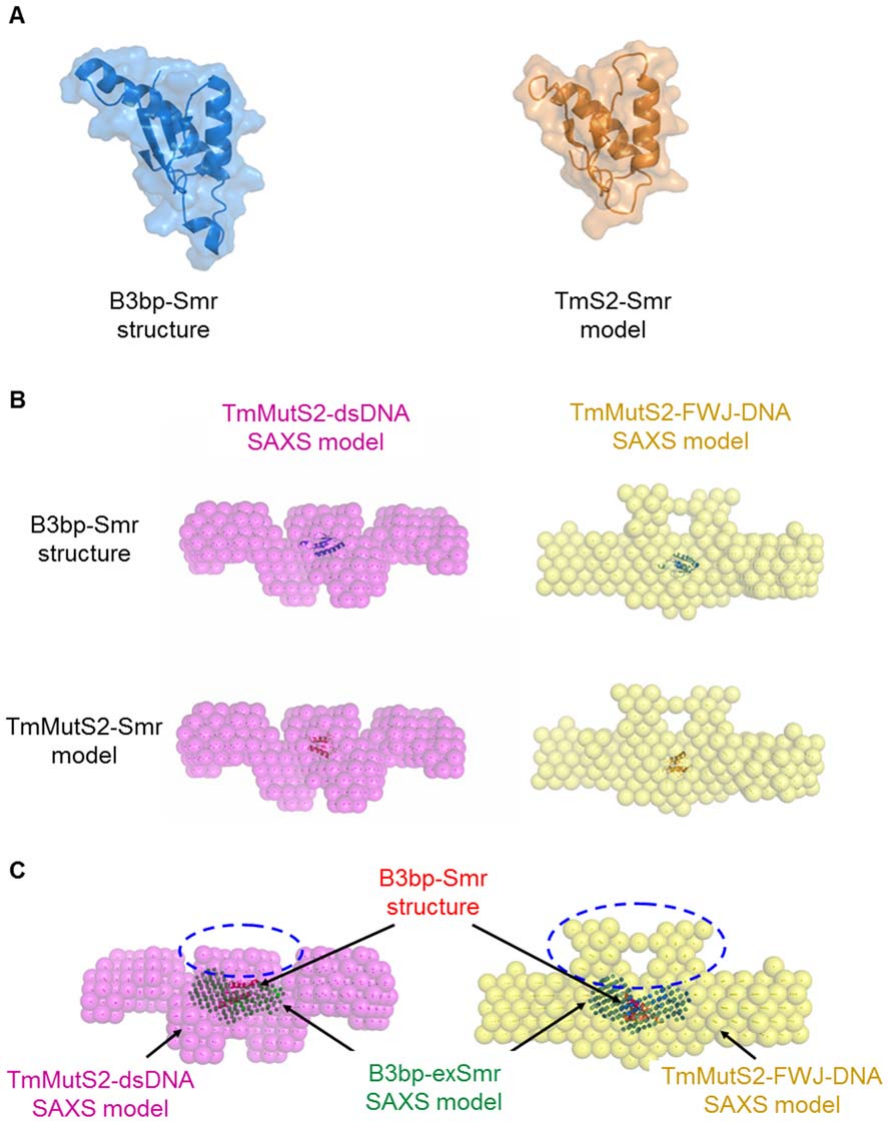
Superimpositions among the TmMutS2-dsDNA/-FWJ-DNA SAXS models and the Smr structure/model. (**A**) The left structure is the monomeric Smr domain of *H. sapiens* B3bp (B3bp-Smr), and the right model is the predicted monomeric Smr domain of TmMutS2 (TmS2-Smr). (**B**) Superimpositions of the TmMutS2-dsDNA/-FWJ-DNA SAXS model to the B3bp-Smr structure and the TmS2-Smr model. (**C**) Superimpositions of the TmMutS2-dsDNA/-FWJ-DNA SAXS model to the B3bp-exSmr SAXS model (green mesh). The B3bp-Smr structure (red) fits well with the B3bp-exSmr SAXS model.

In addition to ATPase domain analysis, the Smr domain of TmMutS2 was also characterized using the sequential homology of the Smr domain (683–757) of TmMutS2 with the Smr domain (1691–1770) of human B3bp (Figure S8). Like the preceding modeling, the TmMutS2-Smr domain was theoretically modeled from the high-resolution structure of B3bp-Smr (PDB No. 2D9I), as shown in Figure 8A. Using the TmMutS2-Smr model and the B3bp-Smr structure, the superimposition between TmMutS2-DNA (along with the dsDNA and FWJ-DNA complexes) and SAXS models was carried out (Figure 8B). Although the sizes of the B3bp-Smr structure and SAXS models seem to be different, they are similar in size. The final overlay indicated that the TmMutS2 SAXS models fit well with the respective B3bp-Smr structure and the TmMutS2-Smr model. The final fineness values for the SAXS templates and structures were lowered compared to their initial values (Table S1). In the superimposed models, both B3bp-Smr and TmMutS2-Smr are localized to the central region of the TmMutS2-DNA SAXS models, indicating that the central region of TmMutS2 is perhaps conserved but that the N-terminal region is very flexible. Interestingly, the ATPase domain (residue 310-502) of TmMutS2 is also localized to the middle location as previously described (Figure 7B). To explore the central region in more detail, these regions of the TmMutS2-DNA SAXS models were superimposed onto the B3bp-exSmr SAXS model. The good fit with the B3bp-Smr structure (Figure 8C, green mesh region) indicates that TmMutS2 has conserved domains, such as the C-terminal and DNA-binding domains. These results reveal that the addition of dsDNA and FWJ-DNA to TmMutS2 causes conformational changes in TmMutS2 (Figure 8C, circle dash-lines). These results were in accordance with the DNA-binding activities experiments (Figure 1C).

## Discussion

Studies on the conformational alterations of proteins containing conserved domains, such as ATPase and DNA-association domains, provide important clues for biological mechanisms [40]. To validate the conformational changes of TmMutS2, proteolytic digestion was carried out. As shown in Figure 1D, while the conformational changes of TmMut2 were weakly induced in the presence of FWJ-DNA, the addition of nucleotides resulted in the decrease of digested TmMutS2 fragments. These results indicate that the structure of TmMutS2 is altered through nucleotide binding. A similar phenomenon has been previously reported for the MutS-mismatched DNA complex that presented defective proteolysis with trypsin rather than apo-MutS [40]. Thus, these results imply that the TmMutS2-nucleotide complexes may have a more compact structure than apo-TmMutS2 because of their flexibility, which coincides with the result of the SEC experiment (Figure 1E). In addition, N-terminal amino acid sequencing analysis on digested TmMutS2 revealed that the largest fragment was the 118–757 residues and that the digested fragment from the TmMutS2 N-terminal was near 14 kDa. This implies that the N-terminal of the conserved DNA binding domain is not mainly engaged in direct DNA binding, having the high flexibility. Biochemical substantiations of the flexibility of the N-terminal coincide exactly with SAXS models of TmMutS2-DNA complexes, which will be debated in discussion for SAXS results.

While dimerization is a common feature of many endonucleases, including restriction enzymes and Holliday junction-related enzymes [41], [42], monomeric endonucleases also exist and are similar in size to *E. coli* MutH [43]. The TmMutS2-Smr domain used in this study was composed of 130 amino acid residues. Interestingly, whereas TmMutS2 had a dimeric structure (Figure 1A, 1B, and 1E), TmMutS2-Smr with high homology to *T. thermophilus* MutS2-Smr [18] was a monomer with no multimeric form as represented in Figure S9. The experimentally obtained molecular weight (approximately 18 kDa) of TmMutS2-Smr was comparable to the calculated molecular weight (15 kDa) of monomeric TmMutS2-Smr. Such monomeric TmMutS2-Smr shows the higher nicking endonuclease activity rather than dimeric apo-TmMutS2 (Figure 2B). Although dimerization results in a decrease of the endonuclease activity, specificity of the protein for substrates can be relatively enhanced. Therefore, it is expected that the dimeric conformation of TmMutS2 is the key feature for controlling the activity as well as specificity.

MutL, one of the major proteins associated with the MMR event, interacts with MutS [1]–[4], [44]. It also stimulates the nicking endonuclease activity of MutH in the *E. coli* MMR system [1], [3], [4], in which the C-terminal region of MutL plays an important role in the activation of MutH [45]. Importantly, this implies that a protein having a relative sequence homology with the C-terminal regions of MutL in the bacterial systems lacking MutH [17] may have a complementary interaction with MutL. Thus, there is a possibility that the C-terminal regions of MutL interact with the Smr domain of MutS2. Non-specific nicking endonuclease activity of TmMutS2, that is dependent on diverse factors such as temperature, amount and type of cations, and the types of nucleotides, increased following the addition of TmMutL (Figure 3A). These results suggest that interactions between TmMutS2 and TmMutL successfully improve the nicking endonuclease activity of TmMutS2. However, ADPnP negatively affected the nicking endonuclease activity of TmMutS2, irrespective of the presence of TmMutL (Figure 2C, 3B), indicating that the ATPase domain is close to the Smr domain and far from the binding site of TmMutL. This proximity will be further discussed in the SAXS part.

In the various eukaryotic DNA repair mechanisms, nicking endonucleases are involved in the excision of damaged DNA as well as the recognition of specific nicked sites in strand displacement reactions [46]. Recent results demonstrate that the bacterial and eukaryotic MutS2 sequence homologues have the ability to identify damaged DNAs and bind to it [6], [15], [17]. The sequence alignment of MutS2 homologues suggests that the Smr domain of MutS2 can introduce a nick in DNA, and then the nick is extended by other exonucleases, resulting in undesirable strand exchanges and the early termination of recombination. Therefore, the mutation frequency can be influenced by MutS2 due to its endonuclease activity. In practice, the incorporation of the exogenous the *TmmutS2* gene into the *E. coli* chromosome containing RecA yielded the important result that TmMutS2 inhibited the RecA-mediated recombination reaction *in vivo* and *in vitro* (Figure 4). Interestingly, the DNA strand-exchange reaction was further negatively impacted by the addition of TmMutL, which provided the surprising clue that TmMutS2-TmMutL interaction might be an essential or additional condition for DNA homologous recombination (Figure 4C) similar to the MutS-MutL complex [33]. In the human recombination system, the MSH4-MSH5 heterodimer interacts with the MutLα heterodimer that has an endonuclease function (MLH1-PMS2 in human and MLH1-PMS1 in yeast) [47]. However, sequence analysis of TmMutS2 indicates that the protein lacks any known nuclease motif present in the MutLα heterodimer. Although TmMutS2 shows an unlinked relationship by primary sequence analysis, the biological functions of eukaryotic MutS2 sequence homologues in DNA recombination system may be analogous to the DNA binding and endonucleolytic activities of TmMutS2.

In addition to the biochemical characterization of TmMutS2, SAXS offered structural information on the protein and made it possible inspect the structural features of TmMutS complexes with nucleotides and DNA. Through the modeling of the various complexes, the TmMutS2-ADP model was confirmed to have a compacted form contrary to the extended structures of TmMutS2-DNAs models and in accordance with biochemical results (Figure 6). Therefore, it is expected that this structural compactness has the possibility of suppressing the nicking endonuclease activity of TmMutS2.

In the case of the superimposition of the ATPase domain (residue 310–502) to the SAXS models, the domain was localized in the center of TmMutS2. Additionally, the superimpositions among the TmMutS2-ADPnP SAXS model and the TaqMutS-ATPase structure/TmMutS2-ATPase model indicate that dimer interfaces maybe exist in the middle region (Figure 7B).

The TmMutS2-FWJ-DNA SAXS model had a large central cavity in the superimposed region and retained a more globular form compared to the other SAXS models (Figure 8B). Also, superimposition of TmMutS2-FWJ-DNA or -dsDNA SAXS models to the B3bp-Smr structure or the TmMutS2-Smr model exhibits an intrinsically flexible N-terminal that would allow for a certain conformational variability in the apo-TmMutS2 and TmMutS2-nucleotides SAXS models (Figure 6). Particularly, TmMutS2-FWJ-DNA represents the most elongated form, implying that dimeric interactions of the protein enhance the binding affinity for DNA substrates. This result certainly agrees with the biochemical experiments as previously mentioned. Therefore, it is clear that the N-terminal domain of TmMutS2 plays an important role in efficient binding for various types of DNA via conformational transitions. Additionally, the superimposition of the TmMutS2-DNA model to the B3bp-exSmr SAXS model reveals that TmMutS2 retains the structurally conserved Smr domain in the C-terminal and the DNA-binding region in the large central cavity (Figure 8C). This feature is in accordance with the result of previous biochemical assay that ADPnP inhibits the nicking endonuclease activity of TmMutS2. Since both the ATPase and the Smr domains are located in the center of TmMutS2, ADPnP binding interrupts the endonuclease activity of the Smr domain.

Diverse MutS homologues recognize specific DNA substrates such as mismatched DNAs, bulged loops, oxidative-modified bases, and FWJ-DNAs. Besides these DNA-protein interactions, endonucleolytic activity is also required for DNA repair mechanisms [45]. TmMutS2 exhibited non-specific DNA-binding activity and nicking endonuclease activity. In addition, TmMutS2 interacted with TmMutL and regulated the RecA-mediated DNA exchange reaction similar to the MutS2 of other species [15], [17], [20]. Therefore, bacterial MutS2 including TmMutS2 may bind to random DNA, particularly cross-over DNA, and affect recombination reactions. Additionally, the results from our investigations demonstrate that various events related to TmMutS2 and the structure of TmMutS2 can be affected by binding with nucleotides and DNA. The SAXS modeling method was very useful for inspecting the other intermediate conformations of the protein complexes and for further demonstrations of the structure of the partial domains such as ATPase and DNA-binding domains. This amalgamation of biochemical- and SAXS analysis offered valuable information for conformational transitions of TmMutS2 and substantiated various experimental results.

At present, to explore the structural and biochemical characteristics of TmMutS2 in more detail, our group has been continuously trying to determine the high-resolution structure of TmMutS2 using both X-ray crystallographic and the SAXS-based methods. The experimental findings described in this study may help to understand the multiple functions and structural characteristics of TmMutS2. It is expected that these investigations will provide critical insights into the functionalities of the prokaryotic and eukaryotic MutS2 sequence homologues involved in DNA homologous recombination, as well as aid in perfecting comprehension of the accurate and detailed functions of MutS2.

## Materials and Methods

### Cloning, Over-expression, and Purification of TmMutS2

The *Tmmut*S2, *Tmmut*L, and *Tmrec*A genes were isolated from *T. maritima* MSB8 genomic DNA. In addition, the *b3bp-Smr* and *b3bp-exSmr* genes were isolated from *Homo sapiens* HeLa cell complementary DNA. All genes were amplified by PCR using specific primers as shown in Table S2. The amplified genes were inserted into the bacterial expression vector pET-28aTEV (Novagen, Germany) containing the TEV protease recognition site. In addition, the *Tmmut*S2Δ*Smr* and *Tmmut*S2-*Smr* (only Smr domain of TmMutS2) genes were isolated from pET-28TEV-*Tmmut*S2, and then ligated into pET-28aTEV. The clone sequences were confirmed via automated DNA sequencing. The target proteins were over-expressed in BL21 (DE3) cells (Invitrogen, USA) grown in Luria-Bertani (LB) medium containing 50 µg/mL kanamycin at 37°C. When the UV absorbance of the cells at 600 nm reached 0.6, the protein expression was induced by the addition of 0.5 mM isopropyl-thiogalactopyranoside. The induced cells were then kept at 37°C for an additional 6 h. After the cells were harvested by centrifugation, they were resuspended in a lysis buffer (20 mM Tris-HCl, pH 8.0, 500 mM NaCl, 0.5 mM β-mercaptoethanol, 5% glycerol, 1 mM phenylmethyl-sulfonyl fluoride, 1 µg/mL leupeptin, 1 µg/mL aprotinin, and 1 µg/mL benzamidine), and then disrupted by sonication. The lysate was centrifuged at 13000 rpm for 30 min at 4°C, and the supernatant was used for the further experiments. Prior to chromatography, a heat-activation step performed at 65°C for 1 h was employed for only TmMutS2, TmMutL, and TmRecA to remove the endogenous proteins extracted from the cells. In the case of B3bp-Smr, supernatants were directly applied to a nickel-nitrilotriacetic acid (Ni-NTA, GE Healthcare, Denmark) affinity column pre-equilibrated with buffer I (20 mM Tris-HCl, pH 8.0, 500 mM NaCl, 0.5 mM β-mercaptoethanol, 5% glycerol, and 5 mM imidazole), and then eluted in buffer I with 300 mM imidazole. His-tag fragments of the purified samples were eliminated by adding TEV protease at room temperature (RT) for 8 h. To remove the untreated His-tagged proteins and the cleaved His-tag fragments, the samples were reloaded onto the Ni-NTA affinity column, and the target proteins were obtained via flow-through against buffer I with 30 mM imidazole. For further purification, each protein was individually applied to a Heparin HitrapTM HP (TmMutS2, TmMutL, and TmRecA) and a Mono S (B3bp-Smr) ion exchange column (GE Healthcare, Denmark) equilibrated with buffer II (50 mM Hepes-NaOH, pH 7.5, 100 mM NaCl, 1 mM dithiothreitol (DTT), 5 mM MgCl_2_, 0.5 mM ethylenediaminetetraacetic acid (EDTA), and 5% glycerol). They were eluted in buffer II with 1 M NaCl as a linear gradient (flow rate: 1 to 2 mL/min). The final purification was carried out using Superdex 200 HR (TmMutS2, TmMutL, and TmRecA) and Superdex 75 HR (B3bp-exSmr and B3bp-Smr) size exclusion columns (GE Healthcare, Denmark) pre-equilibrated with buffer III (25 mM Hepes-NaOH; pH 7.5; 100 mM NaCl for B3bp-exSmr and B3bp-Smr or 200 mM NaCl for TmMutS2, TmMutL, and TmRecA; 5 mM MgCl_2_; 0.5 mM EDTA; and 5% glycerol). The highly purified proteins were concentrated using an Ultracel Amicon YM-10 (Millipore, USA), and analyzed by sodium dodecylsulfate polyacrylamide gel electrophoresis (SDS-PAGE).

### Sequence Alignment Analysis

The multiple sequence alignments for the TmMutS2-related proteins such as TmMutS2, MutS2 homologues, TmMutS2-Smr, and Smr homologues were performed using CLUSTALW [48] and ESPript version 2.2 (http://espript.ibcp.fr/ESPript/cgi-bin/ESPript.cgi) [49] with the default parameter settings (Figure S1).

### Measurements of ATPase Activity

TmMutS2 was incubated with 750 pmol of α-^32^P-labeled ATP at 37 and 60°C for 30 min in a reaction solution containing 0.1 M KCl, 50 mM Tris-HCl (pH 8.0), and 5 mM MgCl_2_. One microliter of each reaction mixture was spotted onto a PEI-TLC plate (Figure S2). Labeled ATP and ADP were separated by developing the TLC plate in an incubation solution containing 1 M formic acid and 0.4 M LiCl [50] before detection using a bio-imaging analyzer (FLA-2000, FUJIFILM). ATP hydrolysis kinetic data were obtained for various concentrations of TmMutS2 (0.7, 1.2, and 2 µM) with 500 µM ATP. To calculate the specific ATPase activity of TmMutS2, a fluorometric real-time assay was carried out [51]. The reaction was conducted in a solution consisting of 0.1 M KCl, 50 mM Tris-HCl, pH 8.0, 5 mM MgCl_2_, 90 µM NADH, 0.9 mM PEP, 5.3 U/mL pyruvate kinase, 7.5 U/mL lactate dehydrogenase, 0.5 mM ATP, and 400 nM TmL. The reaction was continuously monitored at 340 nm using a spectrophotometer (Shimadzu UV1601, Germany). The influence of stability of TmMutS2 to ATPase activity was monitored using a DLS instrument (Zetasizer Nano Malvern, UK). It was performed with TmMutS2 in buffer III at both 37°C and 60°C for 0, 30, 60, and 90 min (Figure S2).

### Scanning Probe Microscopy Observation

The highly purified TmMutS2 was spread on a fresh circular mica sheet (9.9 diameters; Ted Pella Inc., USA). A series of droplets was gently dispensed on the central mica surface by pipetting [52]. Sample mounting was conducted in approximately 1 min intervals as follows: 50 µL of double-distilled (dd) H_2_O for pre-rinsing, 50 µL of 100 mM MgCl_2_ for supporting affinity of the proteins, 50 µL of ddH_2_O for rinsing, 10 µL of TmMutS2 solution, and then three applications of 50 µL of ddH_2_O for post-rinsing of the mica surface. After the sample was mounted, the mica sheet was air-dried, and then inspected using SPM as shown in Figure 1A.

### Analytical Ultracentrifugation

To verify the homogeneity of TmMutS2, the sedimentation velocity (SV) measurements were performed using an Optima XL-A analytical ultracentrifuge (BECKMAN, USA) equipped with an An60Ti rotor and a photoelectric scanner at 20°C as previously described [30]. The protein was injected into a double-sector cell with a 12-mm Epon centerpiece and quartz windows. The rotor speed was 40000 rpm, and the running time was 24 h. All parameters for the SV analysis were adjusted to standard conditions (water at 20°C). The data were analyzed by the C(s) method [31] using the ULTRASCAN program (Figure 1B).

### DNA Binding Assay

Oligonucleotides were prepared as shown in Table S3 according to previously reported DNA sequences (1: blunt duplex, 2: 5′-overhang, 3: FWJ, 4: flayed) [4], followed by heating to 95°C, and then cooling to 25°C for 6 h to anneal each strand. A polynucleotide kinase (TAKARA, Japan) was used to label the 5′-end of various DNAs (10 nM) with [γ–^32^P]ATP (30 nM). Labeled DNA was purified by phenol/ethanol precipitation. TmMutS2 (0, 0.5, 1.0, and 2.0 µM) was incubated with unlabeled and labeled DNAs (1 µM) at 37°C for 1 h, separately in binding buffer (25 mM Hepes-NaOH, pH 7.5, 200 mM NaCl, and 0.5 mM EDTA). To observe the effect of ATP, 1 mM ATP was combined with TmMutS2 (2.0 µM). All results were analyzed using an electrophoretic mobility shift assay with a BAS 2000 bio-image analyzer, and quantified using the Image Gauge program (Figure 1C).

### Proteolytic Digestion

Limited proteolytic analysis using chymotrypsin was carried out in reaction buffer (25 mM Hepes-NaOH, pH 7.5, 200 mM NaCl, 5 mM MgCl_2_, and 0.5 mM EDTA) at 37°C to verify conformational discrepancies among the TmMutS2 complexes. The protease-to-TmMutS2 ratio was optimized to 1:1000 (w/w) for time-resolved digestion with 5 µg of initial TmMutS2. To observe the digestion patterns of TmMutS2, 0.3 mM ADP, 1 mM ADPnP, and 1 µM FWJ-DNA were added to respective proteolytic reaction mixtures. Only FWJ-DNA was employed for proteolytic analysis due to its high binding affinity with TmMutS2. Aliquots of the each total reaction mixture were withdrawn at specific time intervals. At each time point, 16 µL of each reaction mixture was combined with 4 µL of protein-staining buffer, followed by boiling and analysis by 15% SDS-PAGE (Figure 1D).

### Size exclusion Chromatographic Analysis

TmMutS2 (10 µM) was pre-incubated with two types of nucleotides, 1 mM ADP and 1 mM ADPnP at 37°C for 2 h. Apo- and complex-protein forms along with nucleotides were loaded into the Superdex 200 HR size exclusion column pre-equilibrated with buffer III at 20°C to validate the conformational transitions of the TmMutS2 complexes. Eluted proteins were characterized in terms of K_av_ value: K_av_ = (V_e_–V_o_)/(V_t_–V_o_), where V_e_ is the elution volume of each protein, V_o_ is the void volume of the column, and V_t_ is the total bed volume determined by the elution of blue dextran 2000 (Sigma). Molecular weight standard proteins (Sigma, USA), apo ferritin (440 kDa), catalase (232 kDa), and bovine serum albumin (BSA, 66 kDa in the monomer form) were used for size calibration (Figure 1E).

### Measurements of Endonucleolytic Nicking Activity

To determine whether TmMutS2 possessed nicking endonuclease activity, fresh pBlueScript KS (II) (Stratagene, USA) plasmid DNA was utilized. First, to isolate the supercoiled circular plasmid DNA form among the several types of DNA, an agarose gel extraction method was employed. Then, to compare nicking endonuclease activities among MutS2 homologues, supercoiled plasmid DNA (1.5 µM, approximately 3 kbp size) was incubated separately with 2 µM of TmMutS2, TmMutS2ΔSmr, TmMutS2-Smr, and another Smr homologue (B3bp-Smr) in 20 mM Tris-HCl (pH 7.5), 150 mM KCl, 10 mM MgCl_2_, and 1 mM DTT at various temperatures ranging from 20°C to 80°C. In addition, various metal ions were added to the TmMutS2 DNA endonucleolytic reactions. The reaction mixtures were incubated at 60°C for 2 h to help establish optimized conditions. Additionally, TmMutS2 was incubated with nucleotides at various concentrations to verify the effect of the nucleotides (Figure 2).

2 µM of TmMutL was incubated with 2 µM TmMutS2 in the presence of 1 mM of nucleotides (ATP, ADP, and ADPnP) to study the effects of TmMutL. Each reaction was quenched by addition of DNA loading buffer (5 mM EDTA, 1% SDS, 50% glycerol, and 0.05% bromophenol blue), and then analyzed by 0.8% agarose gel electrophoresis (Figure 3).

### Spontaneous Mutation Frequency Analysis

The spontaneous mutation frequency of the *E. coli* BL21(DE3) strain was estimated from the observed frequency of rifampicin-resistant mutants. Rifampicin is a bactericide, an inhibitor for the DNA-dependent RNA polymerase. Wild-type BL21(DE3) with only the pET-28aTEV vector, BL21(DE3) with the pET-28aTEV-*Tmrec*A, and BL21(DE3) with pET-28aTEV-*Tmmut*S2 were individually cultured in 3 mL of LB medium at 37°C for 12 h. Each culture was diluted with 150 mL of LB medium, and then shaken at 37°C for 6 h. These cultures were spread on two types of LB plates, rifampicin-free plates or rifampicin plates containing 50 µg/mL rifampicin. The plates were incubated at 37°C for 24 h. The frequencies of rifampicin mutants per 10^8^ cells were calculated from the number of colonies formed on the rifampicin-containing and rifampicin-free LB plates (Figure 4A).

### *In vitro* DNA Homologous Recombination Assay

The reaction mixtures containing 1 µM M13 phage single-stranded DNA, 1 µM SSB, 10 µM TmRecA, 50 mM Hepes-KOH (pH 7.4), 10 mM MgCl_2_, 2 mM ATP, 1 mM DTT, and an ATP generation system (10 mM phosphocreatine and 10 U/mL phosphocreatine kinase) were pre-incubated at 37°C for 10 min [53]. For initializing the DNA strand exchange reaction, 2 µM EcoRI-linearized M13 phage dsDNA (replication form) was added to the mixtures. Various amounts of TmMutS2 were applied, and the mixture was incubated at 37°C for 1 h. The reactions were terminated by the addition of 25 mM EDTA and 0.1% SDS after a 1 h incubation. The DNA from the reaction was analyzed with a 0.8% agarose gel stained with 0.0001% ethidium bromide (Figure 4B).

### SAXS Data Collection and Processing

SAXS data were collected at the BL45XU beamline of the RIKEN Institute, SPring-8 (Japan). To obtain SAXS data for apo-TmMutS2 and its complexes with ADP, ADPnP, linear dsDNA, and FWJ-DNA, 29 µM of TmMutS2 was incubated with 1 mM of nucleotides and 0.15 mM DNA at 45°C for 30 min, respectively. In the case of B3bp-exSmr (residue 1618-1770 of B3bp), 100 µM of the protein was used. The sample buffer consisted of 20 mM Tris-HCl (pH 7.6), 200 mM KCl, 10 mM DTT, 1 mM EDTA, and 5% glycerol. For minimizing aggregation, all reactants were centrifuged for 10 min immediately before data collection. The parameters for X-ray scattering were as follows: incident wavelength, 0.9 Å; photon flux, 2×10^11^ photon/sec; distance between the XR-II+CCD detector and the sample chamber, 2149.7 mm; the detector channel, S = 4πsinθ/λ (2θ: scattering angle, λ: wavelength); the calibration standard, BSA; and the S range of data acquisition was from 0.006 to 0.23 Å^–1^ with 100 s of exposure time.

### SAXS Data Analysis

The initial scattering data, I(S), were scaled and subtracted from the buffer scattering value (Figure 5). Variations of the amplitude were observed in the high S range. To ascertain variations, the scattering data of the diverse conditions were linearly extrapolated. In addition, the R_g_ were calculated using a Guinier approximation as implemented in a PRIMUS program [54], [55]. The Guinier approximation assumes that the intensity at a very small angle is represented as I(S) = I(0)exp(–4p^2^R_g_^2^S^2^/3), where the forward scattering, I(0), is proportional to the molecular weight. About 60% points were selected to calculate the R_g_ values because the limit value of sR_g_ was set as 1.3. The P(R) values were calculated from the entirety of the scattering data using an indirect Fourier transform method as implemented in the GNOM program [56]. The D_max_ and R_g_ values, which were different from the R_g_ calculated by the Guinier approximation, were estimated by the P(R) function. Additionally, R_hydro_ was determined using a DLS analysis. The measurement was carried out in buffer containing 20 mM Tris-HCl (pH 7.6), 200 mM KCl, 10 mM DTT, 1 mM EDTA, and 5% glycerol. The calculation used a Debye-Einstein-Stokes equation: D∝κBT/3ηD (κB, Boltzman constant; T, absolute temperature; η, viscosity of the dispersing medium; D, apparent diffusion coefficient).

### Refined Modeling and Superimposition

*Ab initio* modeling was conducted using the DAMMIN program [27] with default settings, assuming that a particle had an unknown shape bias and two-fold symmetry (Figure 6). DAMMIN represents the particle shape with a collection of densely packed beads inside a sphere with D_max_. Prior to superimposition, the ATPase and Smr domains of TmMutS2 were automatically compared with the defined structures of other proteins deposited in the PDB, and then modeled using SWISS-MODEL ver. 8.05 [57]. The low-resolution shapes of the intact and complex forms of TmMutS2 were aligned with the known structures of TaqMutS-ATPase (PDB No. 1EWQ), B3bp-Smr (PDB No. 2D9I), and the modeled structures of the TmMutS2-ATPase and TmMutS2-Smr domains using SUPCOMB [58] as shown in Figure 7 and 8. Refined rigid body models were generated using PyMOL [59].

## Supporting Information

Figure S1 **Domain and sequence alignments for TmMutS2.** (**A**) The schematic domain representations of TmMutS2, MutS2 homologues, MutS, and the Smr single domain. The conserved domains for all proteins were identified by sequence alignment analysis and are represented by the colored regions. All sequences were obtained from Genbank: TmMutS2, *Thermotoga maritima* MutS2 (NP_229083.1); HpMutS2, *Helicobacter pylori* MutS2 (NP_223283); AtMutS2, *Arabidopsis thaliana* MutS2 homologue (NP_200220); HsMSH4, *Homo sapiens* MutS homologue (MSH4, NP_002431.2); HsMSH5, *Homo sapiens* MutS homologue (MSH5, NP_002432.1); EcMutS, *Escherichia coli* MutS (NP_417213); and EcYdaL, *Escherichia coli* YdaL (NP_415856.1). DNA binding (blue) and ATPase (red) indicate the DNA-binding and ATP-hydrolytic domains, respectively. Mismatch (grey) and Smr (green) indicate the mismatched DNA-binding region and the Smr domain, respectively. (**B**) Sequence alignment of the DNA binding domain among TmMutS2 and other MutS2 homologues. (**C**) Sequence alignment of the ATPase domain among TmMutS2 and other MutS2 homologues. This alignment was performed with the ESPript 2.2 program.
(TIF)

Figure S2 **The purity and ATPase activity of TmMutS2.** (**A**) SDS-PAGE analysis of purified TmMutS2 from a 12.5% (w/v) denaturing polyacrylamide gel. The gel was stained with Coomassie blue. The left lane represents size marker proteins. (**B**) ATP hydrolytic activity of TmMutS2 as determined by PEI-TLC analysis (upper) and the fluorometric real-time method (lower). (**C**) DLS analysis of TmMutS2 at 37°C (left) and 60°C (right) according to time.
(TIF)

Figure S3 **Endonuclease activity test for other homologues of TmMutS2.** (**A**) TmMutS2 (2 µM) and TmMutS2ΔSmr (2 µM) were reacted with only supercoiled circular DNA (1.5 µM) at 37°C. (**B**) Plasmid DNA was reacted with 1 µM of TmMutS2-Smr and B3bp-Smr at 37°C. The nick, l, and sc indicate the nicked, linearized, and closely supercoiled open circular DNA forms, respectively.
(TIF)

Figure S4 **DNA-binding affinity of TmMutS2 in the presence of TmMutL.**
(TIF)

Figure S5 **Endonuclease activity test for TmMutL.** The addition of 1 mM of nucleotides to 2 µM TmMutL. The 0.8% agarose gel indicates that TmMutL has no nicking endonuclease activity and dependence on the nucleotides. The nick and sc indicate the nicked and supercoiled DNA forms, respectively.
(TIF)

Figure S6 **A spontaneous mutation frequency assay using TmmutS2 and TmmutS2ΔSmr genes.** Rif^R^ indicates rifampicin-resistance. The frequency of Rif^R^ (y-axis)/10^8^ cells is quantitatively analyzed by counting the number of spontaneously mutated colonies.
(TIF)

Figure S7 **The Guinier approximations for the SAXS scattering curves.** These plots are for apo-TmMutS2 and its complexes with nucleotides (ADP and ADPnP) and DNA (dsDNA and FWJ-DNA).
(TIF)

Figure S8 **Sequence alignments.** (**A**) Sequence alignment between the TaqMutS-ATPase domain and the TmMutS2-ATPase domain. (**B**) Sequence alignment between the TmMutS2-Smr domain and the B3bp-Smr domain. All alignments were carried out using the ESPript 2.2 program.
(TIF)

Figure S9 **Size exclusion chromatographic and SDS-PAGE analyses for TmMutS2-Smr.** Monomeric TmMutS2-Smr was confirmed using a Superdex 75 HR column calibrated with BSA (66 kDa in monomer), α-chymotrypsin (25 kDa), and aprotinin (6.5 kDa).
(TIF)

Table S1 **The fineness parameters of the superimpositions.** These are for the SAXS models of the TmMutS2 complexes, the TaqS-ATPase structure/TmS2-ATPase model, and the B3bp-Smr structure/TmS2-Smr model.
(DOCX)

Table S2 **List of specific primers used in this study.**
(DOCX)

Table S3 **List of specific primers used in this study.**
(DOCX)
