# Supplementary figures and images for: Characterization of Multi-Functional Properties and Conformational Analysis of MutS2 from Thermotoga maritima MSB8
Source: PLoS One. 2012 Apr 24;7(4):e34529. doi: 10.1371/journal.pone.0034529 (PMC3335848; doi:10.1371/journal.pone.0034529)

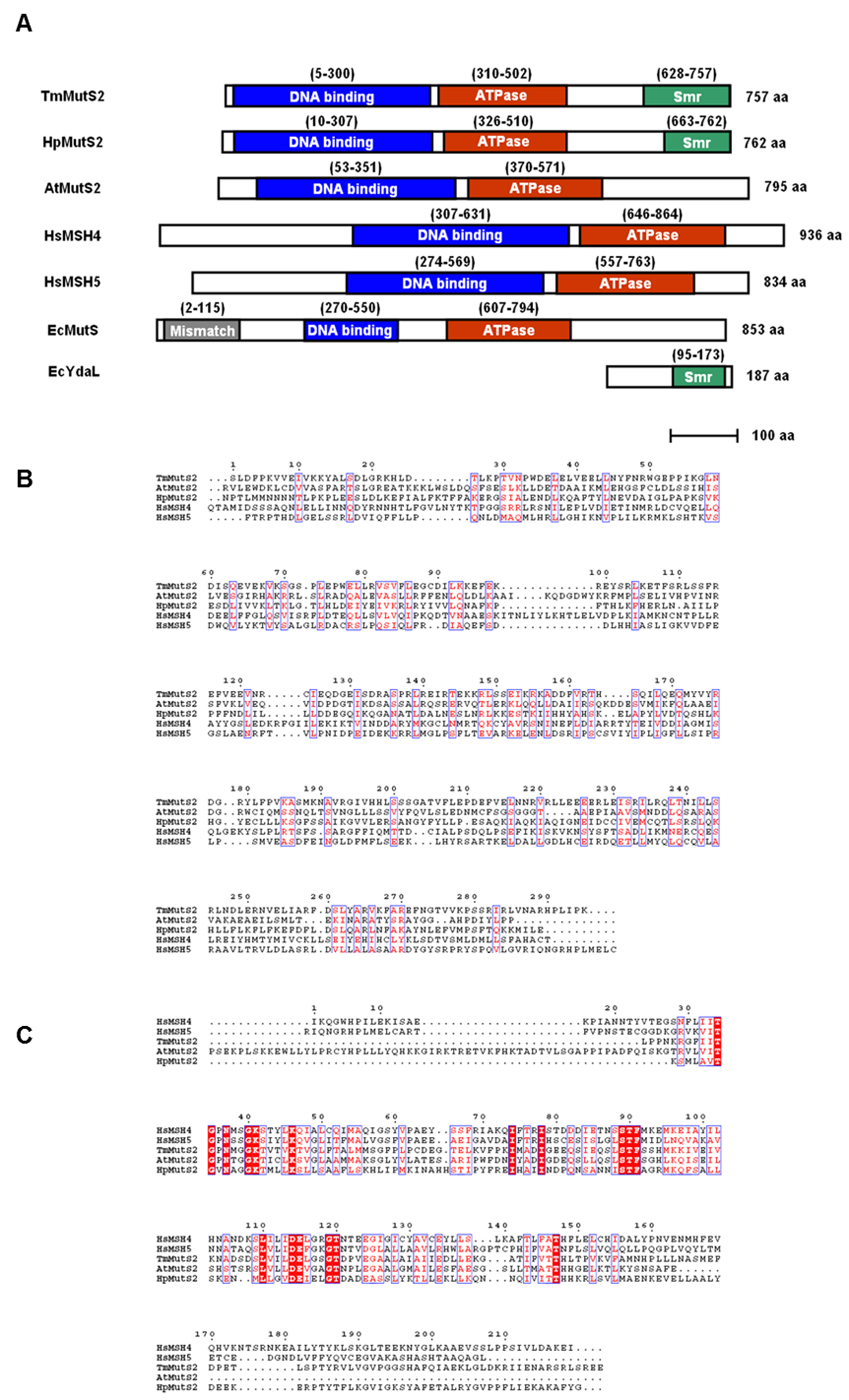

Supplement: Figure S1 — Domain and sequence alignments for TmMutS2. (A) The schematic domain representations of TmMutS2, MutS2 homologues, MutS, and the Smr single domain. The conserved domains for all proteins were identified by sequence alignment analysis and are represented by the colored regions. All sequences were obtained from Genbank: TmMutS2, Thermotoga maritima MutS2 (NP_229083.1); HpMutS2, Helicobacter pylori MutS2 (NP_223283); AtMutS2, Arabidopsis thaliana MutS2 homologue (NP_200220); HsMSH4, Homo sapiens MutS homologue (MSH4, NP_002431.2); HsMSH5, Homo sapiens MutS homologue (MSH5, NP_002432.1); EcMutS, Escherichia coli MutS (NP_417213); and EcYdaL, Escherichia coli YdaL (NP_415856.1). DNA binding (blue) and ATPase (red) indicate the DNA-binding and ATP-hydrolytic domains, respectively. Mismatch (grey) and Smr (green) indicate the mismatched DNA-binding region and the Smr domain, respectively. (B) Sequence alignment of the DNA binding domain among TmMutS2 and other MutS2 homologues. (C) Sequence alignment of the ATPase domain among TmMutS2 and other MutS2 homologues. This alignment was performed with the ESPript 2.2 program. (TIF) [file pone.0034529.s001.tif]

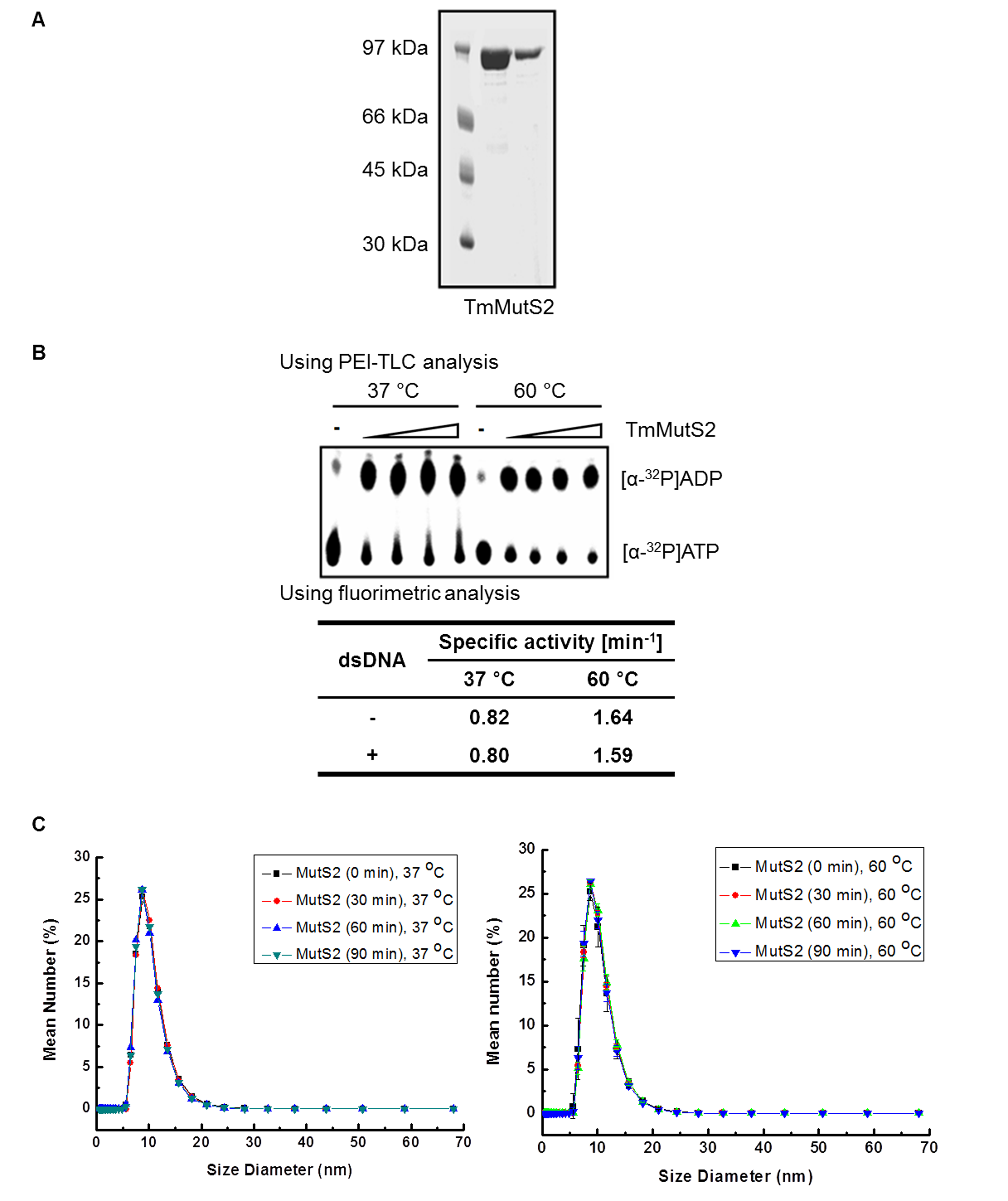

Supplement: Figure S2 — The purity and ATPase activity of TmMutS2. (A) SDS-PAGE analysis of purified TmMutS2 from a 12.5% (w/v) denaturing polyacrylamide gel. The gel was stained with Coomassie blue. The left lane represents size marker proteins. (B) ATP hydrolytic activity of TmMutS2 as determined by PEI-TLC analysis (upper) and the fluorometric real-time method (lower). (C) DLS analysis of TmMutS2 at 37°C (left) and 60°C (right) according to time. (TIF) [file pone.0034529.s002.tif]

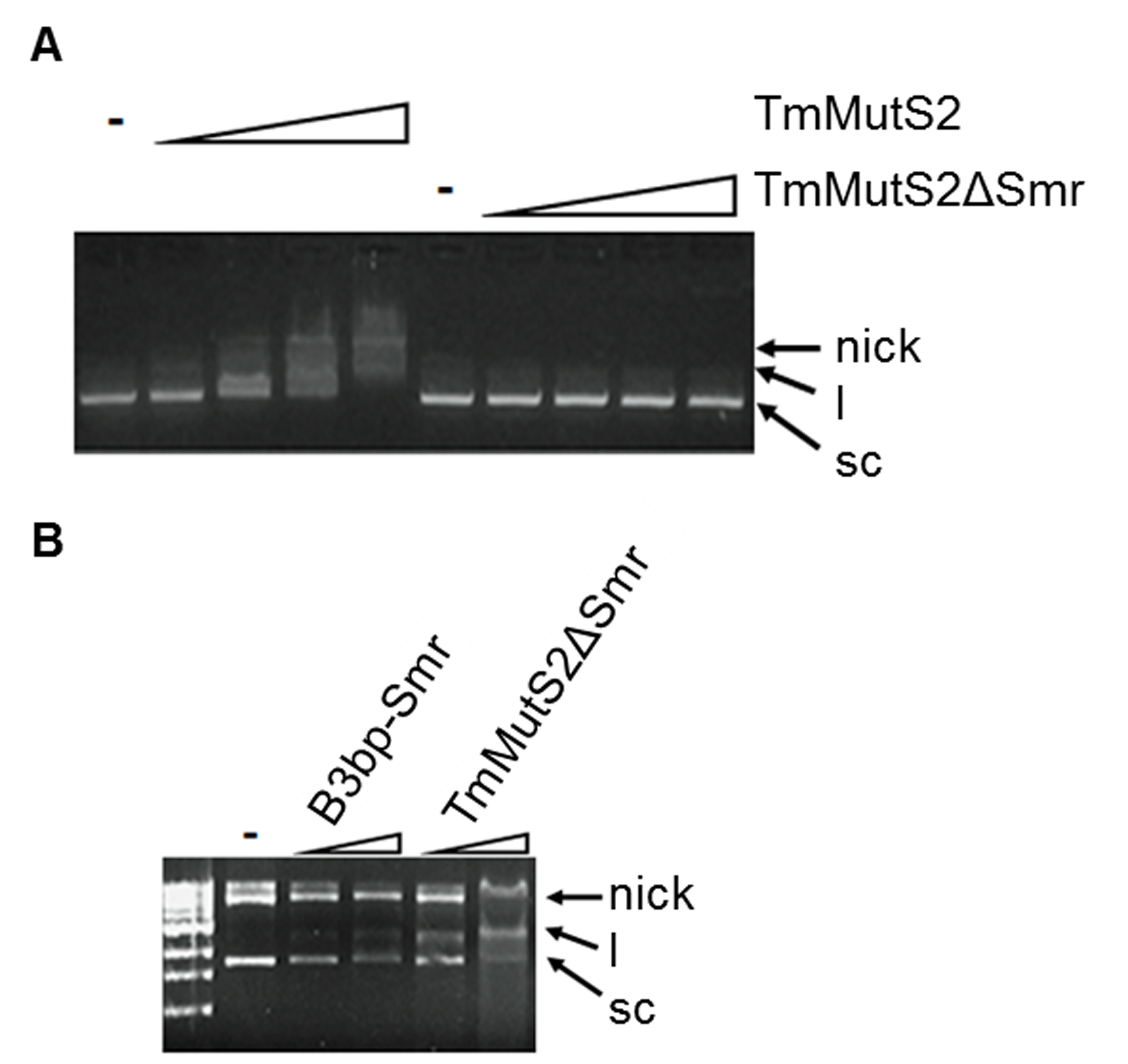

Supplement: Figure S3 — Endonuclease activity test for other homologues of TmMutS2. (A) TmMutS2 (2 µM) and TmMutS2ΔSmr (2 µM) were reacted with only supercoiled circular DNA (1.5 µM) at 37°C. (B) Plasmid DNA was reacted with 1 µM of TmMutS2-Smr and B3bp-Smr at 37°C. The nick, l, and sc indicate the nicked, linearized, and closely supercoiled open circular DNA forms, respectively. (TIF) [file pone.0034529.s003.tif]

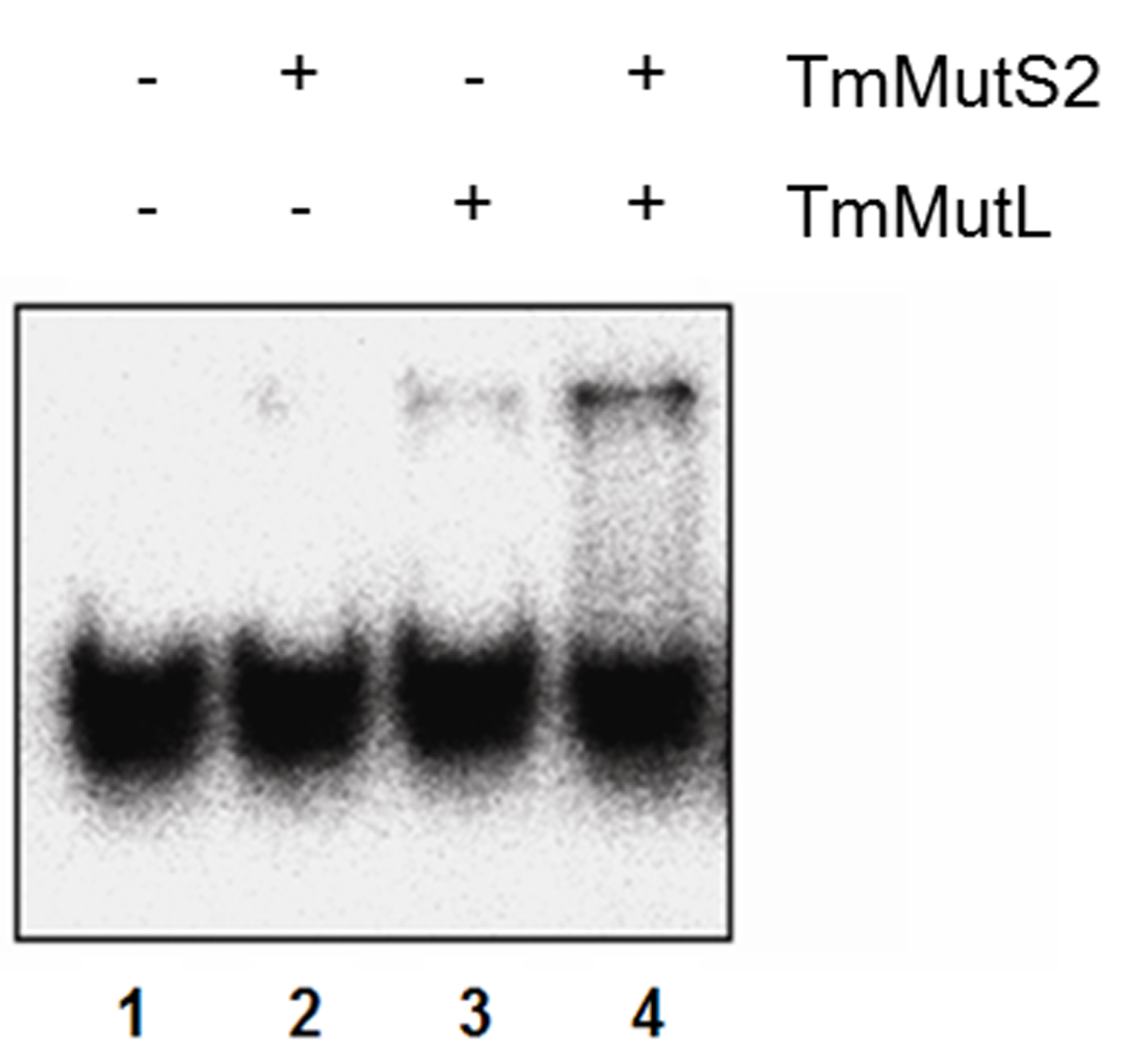

Supplement: Figure S4 — DNA-binding affinity of TmMutS2 in the presence of TmMutL. (TIF) [file pone.0034529.s004.tif]

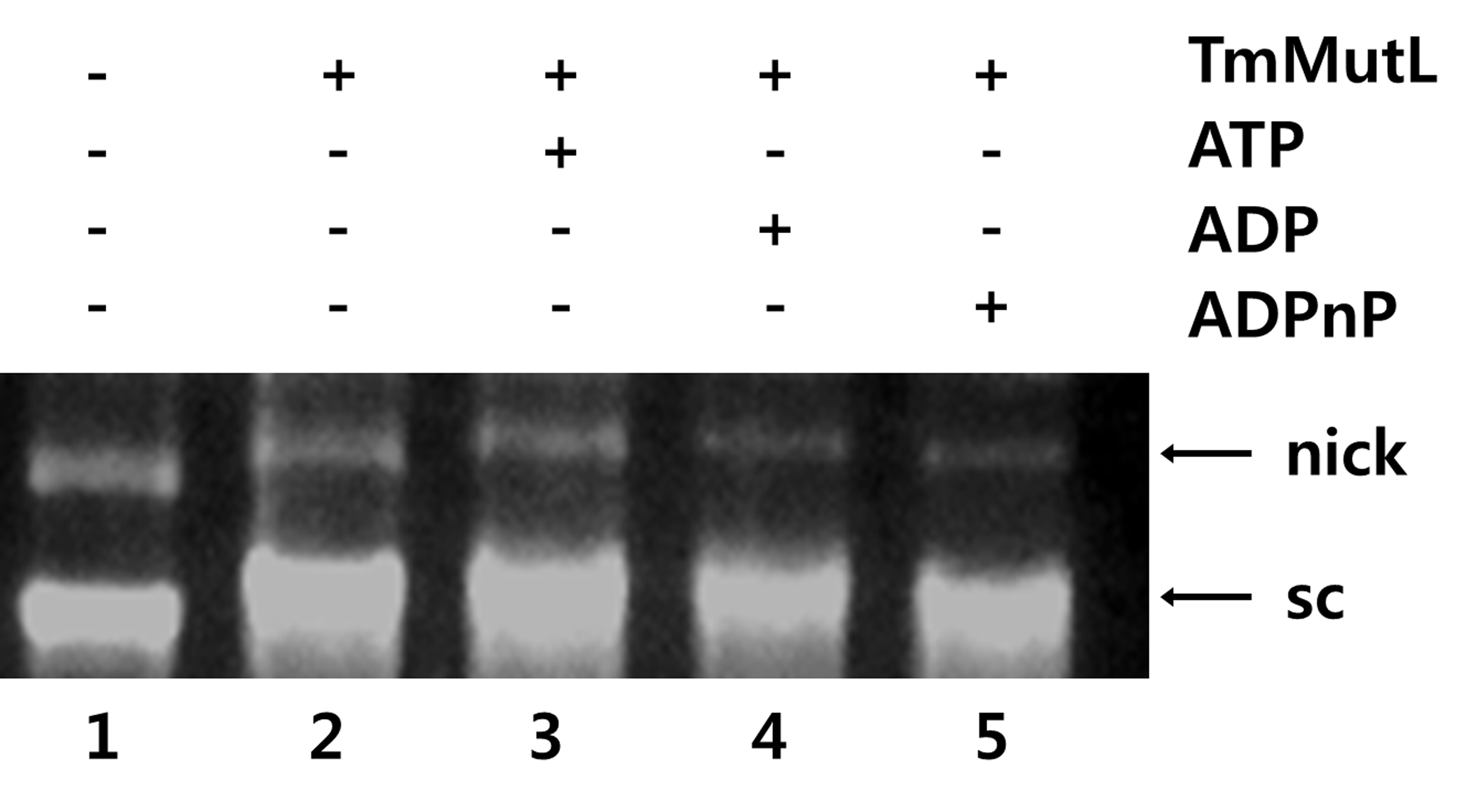

Supplement: Figure S5 — Endonuclease activity test for TmMutL. The addition of 1 mM of nucleotides to 2 µM TmMutL. The 0.8% agarose gel indicates that TmMutL has no nicking endonuclease activity and dependence on the nucleotides. The nick and sc indicate the nicked and supercoiled DNA forms, respectively. (TIF) [file pone.0034529.s005.tif]

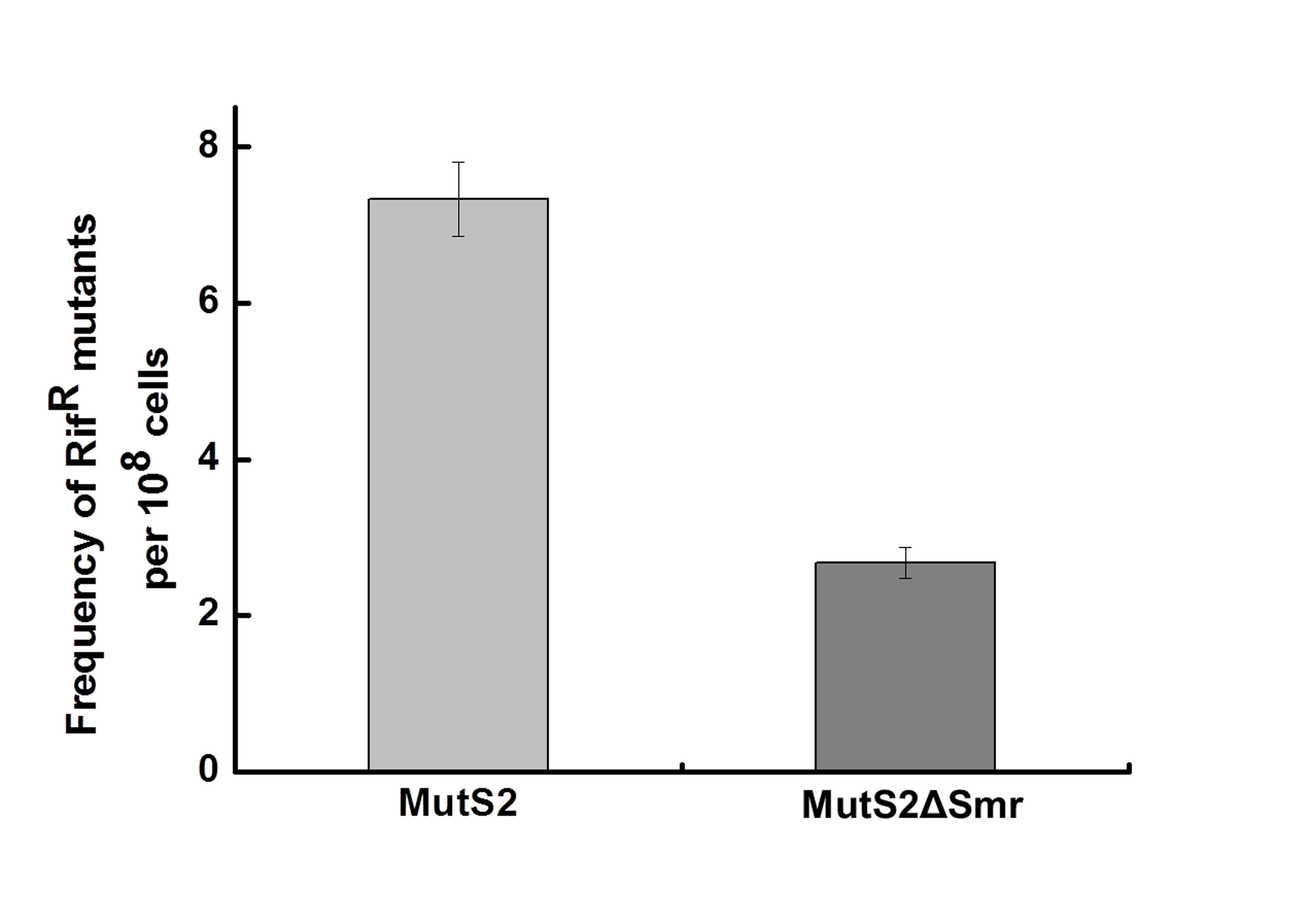

Supplement: Figure S6 — A spontaneous mutation frequency assay using TmmutS2 and TmmutS2ΔSmr genes. RifR indicates rifampicin-resistance. The frequency of RifR (y-axis)/108 cells is quantitatively analyzed by counting the number of spontaneously mutated colonies. (TIF) [file pone.0034529.s006.tif]

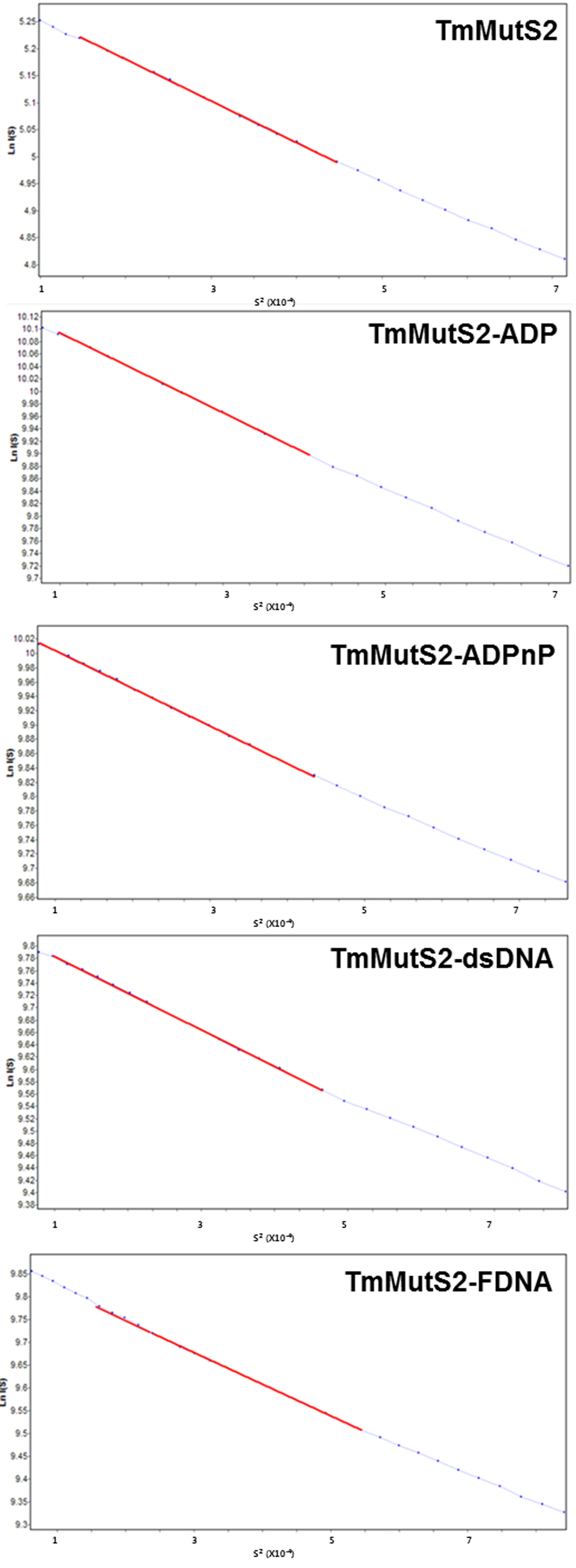

Supplement: Figure S7 — The Guinier approximations for the SAXS scattering curves. These plots are for apo-TmMutS2 and its complexes with nucleotides (ADP and ADPnP) and DNA (dsDNA and FWJ-DNA). (TIF) [file pone.0034529.s007.tif]

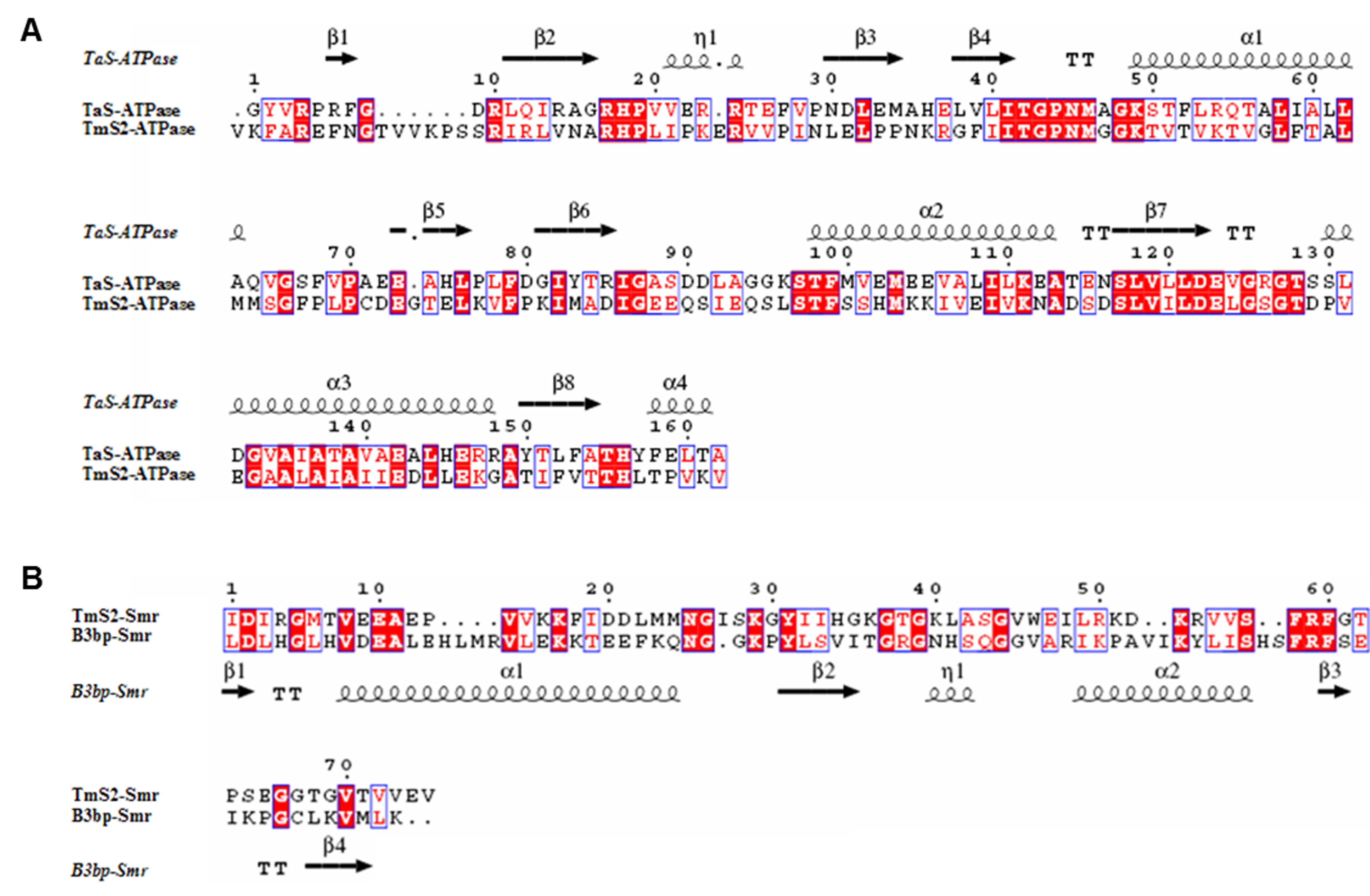

Supplement: Figure S8 — Sequence alignments. (A) Sequence alignment between the TaqMutS-ATPase domain and the TmMutS2-ATPase domain. (B) Sequence alignment between the TmMutS2-Smr domain and the B3bp-Smr domain. All alignments were carried out using the ESPript 2.2 program. (TIF) [file pone.0034529.s008.tif]

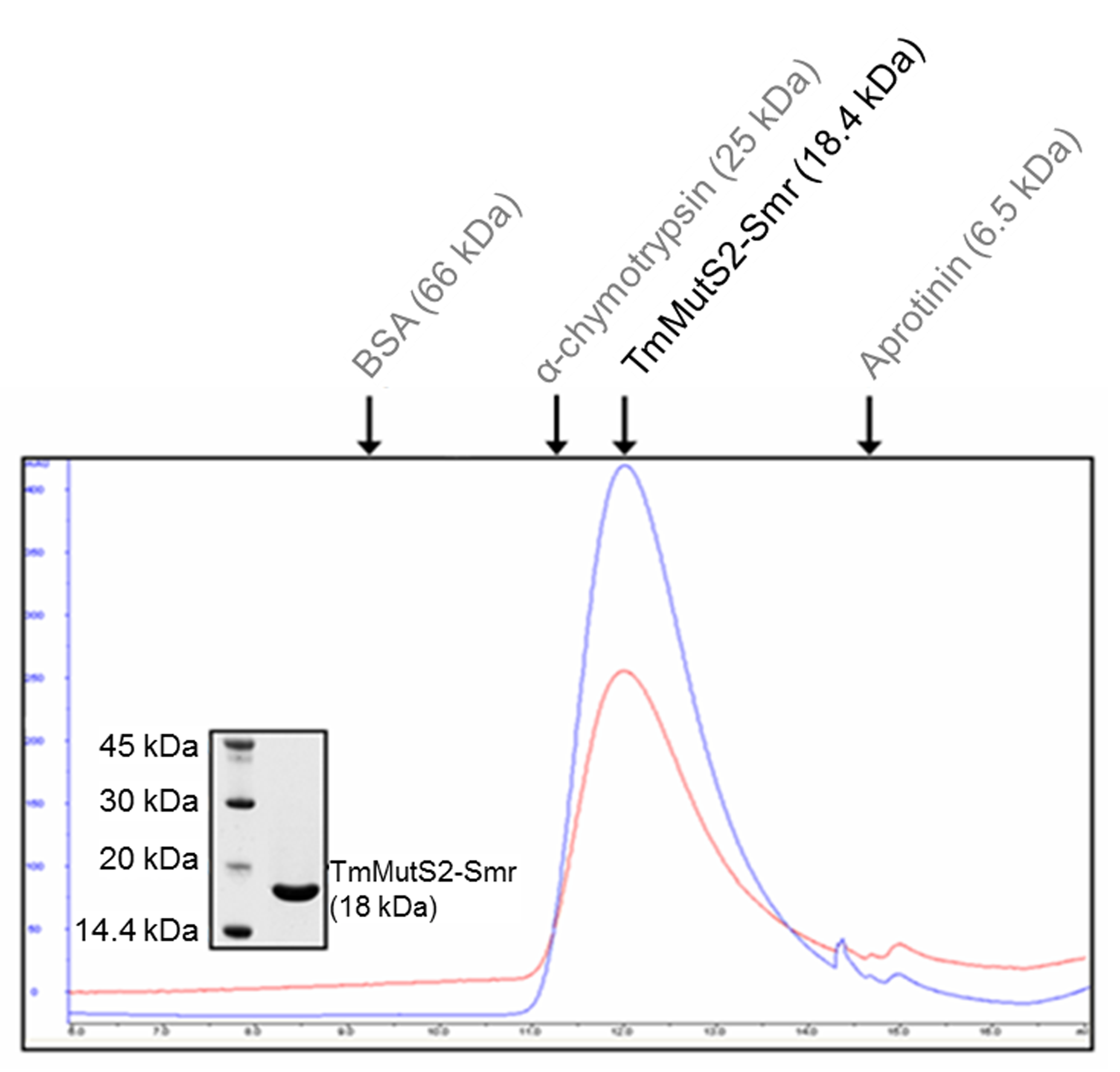

Supplement: Figure S9 — Size exclusion chromatographic and SDS-PAGE analyses for TmMutS2-Smr. Monomeric TmMutS2-Smr was confirmed using a Superdex 75 HR column calibrated with BSA (66 kDa in monomer), α-chymotrypsin (25 kDa), and aprotinin (6.5 kDa). (TIF) [file pone.0034529.s009.tif]
