# Supplementary material for: Characterization of Multi-Functional Properties and Conformational Analysis of MutS2 from Thermotoga maritima MSB8
Source: PLoS One. 2012 Apr 24;7(4):e34529. doi: 10.1371/journal.pone.0034529 (PMC3335848; doi:10.1371/journal.pone.0034529)
Supplement: Table S1 — The fineness parameters of the superimpositions. These are for the SAXS models of the TmMutS2 complexes, the TaqS-ATPase structure/TmS2-ATPase model, and the B3bp-Smr structure/TmS2-Smr model. (DOCX) [file pone.0034529.s010.docx]

**Table S1**

| Types | Fineness | |  | Types | Fineness | |
| --- | --- | --- | --- | --- | --- | --- |
|  | Initial | Final |  |  | Initial | Final |
| TmMutS2-ADPnP | 18.09 | 10.20 |  | TaqS-ATPase | 3.96 | 1.37 |
|  | 18.09 | 10.20 |  | TmS2-ATPase | 3.24 | 1.38 |
| TmMutS2-dsDNA | 22.52 | 15.40 |  | B3bp-Smr | 2.56 | 1.09 |
|  | 22.52 | 15.40 |  | TmS2-Smr | 2.45 | 1.38 |
| TmMutS2-FWJ-DNA | 25.36 | 17.87 |  | B3bp-Smr | 2.77 | 1.12 |
|  | 25.36 | 17.87 |  | TmS2-Smr | 2.36 | 1.32 |

**Table S1.** **The fineness parameters of the superimpositions.** These are for the SAXS models of the TmMutS2 complexes, the TaqS-ATPase structure/TmS2-ATPase model, and the B3bp-Smr structure/TmS2-Smr model.
