# Supplementary material for: Characterization of Multi-Functional Properties and Conformational Analysis of MutS2 from Thermotoga maritima MSB8
Source: PLoS One. 2012 Apr 24;7(4):e34529. doi: 10.1371/journal.pone.0034529 (PMC3335848; doi:10.1371/journal.pone.0034529)
Supplement: Table S2 — List of specific primers used in this study. (DOCX) [file pone.0034529.s011.docx]

**Table S2**

| Types | | Sequences (5’ to 3’) |
| --- | --- | --- |
| TmMutS2 | **Forward** | CGCGAATTCGTGGATTATCTCGAATCACTC |
|  | **Reverse** | CCCAAGCTTTCACACTTTCACCTCCACCAC |
| TmMutL | **Forward** | CGCGGATCCGTGGAGAGGTGTTCTGTTTT |
|  | **Reverse** | CCCAAGCTTTTAACGCTCGAAAAATCGGTC |
| TmMutS2ΔSmr | **Reverse** | CCCAAGCTTTCATTTTGCCACGTGTATCG |
| TmMutS2-Smr | **Forward** | CGCGAATTCTCTGTGGGGAAGGTCGTTG |
| B3bp-Smr | **Forward** | GCCGGATCCTTAGACCTCCATGGGC-3′ |
|  | **Reverse** | GCGCTCGAGTTACTTTAGCATGACTTTCAAGC |
| B3bp-exSmr | **Forward** | GCCGGATCCGACTATGATGACTACAGAGCAGA |
| TmRecA | **Forward** | GCCGGATCCATGCCTGAGGAAAAACAGAAA |
|  | **Reverse** | GCGCTCGAGTTAAGAGGCTTCTTCGCC |

**Table S2.** List of specific primers used in this study.
