# Supplementary material for: Characterization of Multi-Functional Properties and Conformational Analysis of MutS2 from Thermotoga maritima MSB8
Source: PLoS One. 2012 Apr 24;7(4):e34529. doi: 10.1371/journal.pone.0034529 (PMC3335848; doi:10.1371/journal.pone.0034529)
Supplement: Table S3 — List of specific primers used in this study. (DOCX) [file pone.0034529.s012.docx]

**Table S3**

| DNA types | Sequences (5’ to 3’) |
| --- | --- |
| Blunt duplex | ACAGCACCAGATTCAGCAATTAAGCTCTAAGCCATCCGCAAAAATGACCTCTTATCAAAAGG |
|  | CCTTTTGATAAGAGGTCATTTTTGCGGATGGCTTAGAGCTTAATTGCTGAATCTGGTGGCTGT |
| 5’-overhang | ACAGCACCAGATTCAGCAATTAAGCTCTAAGCCATCCGCAAAAATGACCTCTTATCAAAAGG |
|  | TCCTTTTGATAAGAGGTCATTTTTGCGGATGGCTTAGAGCTTAATTGC |
| Four-way junction  (FWJ) | GACGCTGCCGAATCCTACCAGTGCCTTGCTAGGACATCTTTGCCCACCTGCAGGTCACCC |
|  | TGGGTGAACCTGCAGGTGGGCAAAGATGTCCTAGCAATGTAATCGTCAAGCTTTATGCCGTT |
|  | ATCGATAGTCGGATCCTCTAGACAGCATGTCCTAGCAAGGCACTGGTAGAATTCGGCAGCGT |
|  | CAACGGCATAAAGCTTGACGATTACATTGCTAGGACATGCTGTCTAGAGGATCCGACTATCGA |
| Flayed | AACGGCATAAAGCTTGACGATTACATTGCTAGGACATGCTGTCTAGAGGATCCGACTATCGA |
|  | TGGGTGAACCTGCAGGTGGGCAAAGATGTCCTAGCAATGTAATCGTCAAGCTTTATGCCGTT |

**Table S3.** List of specific primers used in this study.
